# Motion Artifacts Removal from Measured Arterial Pulse Signals at Rest: A Generalized SDOF-Model-Based Time–Frequency Method

**DOI:** 10.3390/s25216808

**Published:** 2025-11-06

**Authors:** Zhili Hao

**Affiliations:** Department of Mechanical and Aerospace Engineering, Old Dominion University, Norfolk, VA 23529, USA; zlhao@odu.edu

**Keywords:** arterial pulse signals, arterial pulse waveform (APW), harmonics, instant parameters, motion artifacts (MA), respiration rate, respiration modulation, heart rate (HR), time–frequency analysis, single-degree-of-freedom (SDOF), PPG sensors, tactile sensors

## Abstract

Motion artifacts (MA) are a key factor affecting the accuracy of a measured arterial pulse signal at rest. This paper presents a generalized time–frequency method for MA removal that is built upon a single-degree-of-freedom (SDOF) model of MA, where MA is manifested as time-varying system parameters (TVSPs) of the SDOF system for the tissue–contact-sensor (TCS) stack between an artery and a sensor. This model distinguishes the effects of MA and respiration on the instant parameters of harmonics in a measured pulse signal. Accordingly, a generalized SDOF-model-based time–frequency (SDOF-TF) method is developed to obtain the instant parameters of each harmonic in a measured pulse signal. These instant parameters are utilized to reconstruct the pulse signal with MA removal and extract heart rate (HR) and respiration parameters. The method is applied to analyze seven measured pulse signals at rest under different physiological conditions using a tactile sensor and a PPG sensor. Some observed differences between these conditions are validated with the related findings in the literature. As compared to instant frequency, the instant initial phase of a harmonic extracts respiration parameters with better accuracy. Since HR variability (HRV) affects arterial pulse waveform (APW), the extracted APW with a constant HR serves better for deriving arterial indices.

## 1. Introduction

Clinical values of arterial pulse signals measured at the skin surface at rest have been validated by numerous studies over the past several decades [1,2,3,4,5]. PPG sensors and tactile sensors are the two largest types of sensors used in arterial pulse measurement [1,2,5,6,7,8,9]. As compared to tactile sensors, PPG sensors were used much earlier on, and have been ubiquitously used for pulse measurement, due to their convenience [1,5,9,10]. Regardless of the sensor type, a measured pulse signal at rest is always distorted by motion artifacts (MA), which cause a time-varying distance of the sensor relative to an artery [6,7,8,9,10,11,12,13].

An arterial pulse signal is a collection of multiple harmonics of the heart rate (HR) [14,15]. HR is altered by respiration, due to respiratory sinus arrhythmia (RSA), and by other physiological factors (PF) (e.g., emotion, stress, exercise, etc.), due to the autonomic nervous system (ANS) [16,17]. This explains the reason why a measured pulse signal can be used for respiration rate (RR) extraction [16,18]. Moreover, respiration and PF cause their inherent MA [19,20], and thus, an MA in a measured pulse signal varies between physiological conditions (or individuals). Additionally, the fixture of a sensor at an artery also introduces its own MA [6,7,8]. As such, an MA is a combination of the sensor fixture and the individual (or physiological conditions). Due to MA and HR variability (HRV), the true pulse signal in an artery and its measured signal are both non-stationary [10,16,21].

Because of the availability of many datasets on measured pulse signals using a PPG sensor (or PPG signals), the majority of the studies on MA removal are focused on PPG signals [9,10,20,22,23,24,25,26,27,28,29,30]. In the early days of PPG signal measurement, a PPG sensor was only used at the index finger at rest, and MA at rest is relatively low, yielding a pulse signal with less distortion [1]. MA removal was conducted on a PPG signal to extract its arterial pulse waveform (APW), which was further utilized to derive arterial indices, besides HR and RR [1,9,12,13]. In recent years, PPG sensors have also been used at the wrist as wearable sensors for monitoring HR and RR during activities [9,10,19,20,31,32]. High-level MA during activities greatly distorts a PPG signal, and thus a PPG signal during activities is usually not used for extracting APW [10,20,31,32]. Yet, high-level MA even brings inaccuracy to extracted HR and RR [10,20,33,34]. Here, it is worth noting that MA at rest, albeit being low relative to their counterpart during activities, remain a great challenge for the extraction of both HR and APW with the accuracy needed for clinical diagnosis [22,23,24,25,26,27,28,29,30,33,34].

Tactile sensors based on micro/nano-fabrication technology have also been extensively studied for arterial pulse measurement as an affordable and user-friendly alternative to costly and complex medical instruments [6,35,36,37,38,39,40,41,42,43,44]. They are usually used to measure arterial pulse signals at the wrist and the neck [3,6]. Their configuration usually renders them inconvenient as wearable sensors. As such, tactile sensors are mostly used for pulse measurement at rest, and thus suffer low-level MA. The measured pulse signal is used for the extraction of its APW and, consequently, arterial indices based on the features of the APW and its time derivatives [3,6]. Then, even a relatively small distortion in a measured pulse signal from a low-level MA may affect the accuracy in the extracted APW and its derived arterial indices [21].

A large body of literature exists on different techniques for MA removal from PPG signals [1,9,10,11,12,13,14,15,16,19,20,21,22,23,24,25,26,27,28,29,30,31,32,33,34]. Generally speaking, MA removal techniques can be categorized into two types: reference-based and non-reference-based [19,20]. The reference-based technique requires a reference sensor (e.g., an accelerometer or a PPG sensor) to record MA, which is used to remove MA in the concurrently measured PPG signal [19,20,45]. However, MA encountered by a reference sensor differ from those encountered by a PPG sensor, simply because the tissue they sit on is different [21]. The non-reference-based technique is focused on the development of signal-processing techniques to remove MA [19,20,34]. These signal-processing techniques include statistical methods, machine learning, and signal decomposition [19,20]. While statistical methods demand an accurate system model for a PPG signal and MA characteristics and thus involve great computational complexity [20], machine learning leverages data patterns for MA detection and removal, offering flexibility but often requiring substantial labeled datasets [20]. Both statistical methods and machine learning need training on existing PPG signals, prior to their actual usage for the analysis of PPG signals for clinical values.

Signal decomposition amounts to the time–frequency analysis (TFA) of a PPG signal, which directly tackles its non-stationary nature [10,16,46]. Discrete waveform transform (DWT), empirical mode decomposition (EMD), and varying-frequency complex demodulation (VFCDM) all belong to this category [10,16,19,46]. DWT demands a selection of appropriate wavelet functions, which are critical for the accuracy of MA removal and vary with individuals [19,34]. EMD decomposes signals into intrinsic mode functions, but suffers from mode mixing [16]. VFCDM examines the frequency spectrum of a PPG signal, which is used to identify the segment with small distortion for the extraction of HR and RR [10]. Then, MA in the segment with low distortion is not really removed but neglected. In a nutshell, statistical methods, machine learning, and DWT need to adjust to the nature of MA in a PPG signal [19,46]; EMD faces challenges in effectively separating the harmonics in a PPG signal; and VFCDM identifies the segment in a PPG signal with low distortion but fails to remove MA.

This work aims to develop a generalized time–frequency method for MA removal from a measured pulse signal at rest, regardless of the nature of the MA (or physiological condition and sensor fixture). This method is built upon an SDOF model of MA in a measured pulse signal [7,8], where the transmission path from the true pulse signal in an artery to the measured pulse signal by the sensor at the skin surface, or tissue–contact-sensor (TCS) stack, is modeled as a single-degree-of freedom (SDOF) system to fully account for the dynamic behavior of the TCS stack in pulse measurement [7,8,21]. The model reveals that the MA at rest is manifested as baseline drift (<0.7 Hz) and time-varying system parameters (TVSPs) of the TCS stack. Baseline drift is an additive noise and can be easily removed, since its frequency is below the frequency of a measured pulse signal (>1 Hz). Yet, TVSP-generated distortion is a multiplicative noise riding on each harmonic in a measured pulse signal [6,7,8,21]. To remove TVSP-generated distortion, a time–frequency method based on Hilbert vibration decomposition (HVD) was developed previously, but this method allows separating only the first three harmonics for their MA removal [21].

In this work, a generalized SDOF-model-based time–frequency (SDOF-TF) method is developed for MA removal and extraction of APW, HR, and respiration parameters from a measured pulse signal at rest. As compared to the previous related studies, the original contributions of this work include the following: (1) the SDOF model of MA is used for a thorough examination of the effects of the MA at rest (<0.7 Hz) and during activities (>0.7 Hz) and respiration on the instant parameters (i.e., frequency, amplitude, and initial phase) of a measured pulse signal; (2) a generalized SDOF-TF method is developed that allows separating all the harmonics in a measured pulse signal at rest, reconstructing the pulse signal with MA removal, and extracting APW, HR, and respiration parameters; (3) this method is applied to measured pulse signals at rest using a tactile sensor under different conditions (pre-exercise and 1 min and 5 min post-exercise) and PPG signals in different datasets (atrial fibrillation (AF) versus non-AF, critically ill, and during activities) to examine and validate its effectiveness and generalizability; and (4) the instant initial phase is identified for extraction of respiration parameters with better accuracy, as compared to instant frequency, and the amplitudes and initial phases of harmonics in the extracted APW with constant HR are found to serve better as arterial indices.

## 2. Materials and Methods

In pulse measurement, a sensor is pressed against an artery to establish the tissue–sensor contact at the skin surface, so the true pulse signal in the artery can pass the TCS stack and register as the measured pulse signal by the sensor. For simplicity, the SDOF model of the MA in a measured pulse signal is based on the three assumptions: (1) the TCS stack behaves linearly; (2) the true pulse signal is unaffected during measurement and then the arterial wall displacement is the true pulse signal; and (3) the associated transduction of a sensor are neglected and a measured pulse signal is a displacement output.

### 2.1. Arterial Pulse Signal Measurement Using a Tactile Sensor and a PPG Sensor

**Figure 1:**
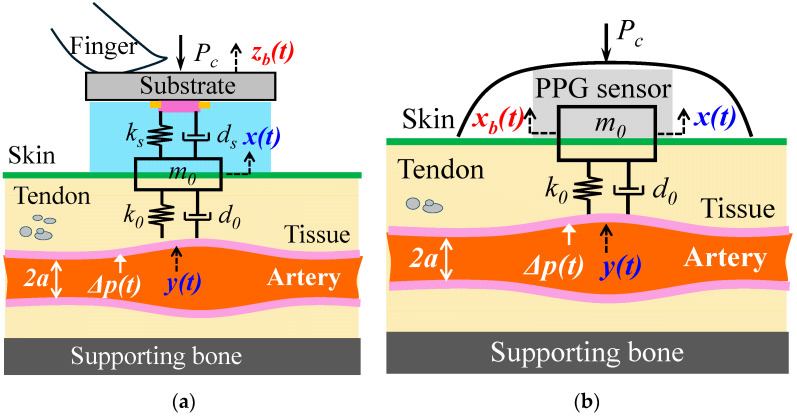
Schematics of arterial pulse measurement using (**a**) a tactile sensor and (**b**) a PPG sensor.

Although tactile sensors take various forms [6], in essence, a tactile sensor contains a microstructure on a substrate and a transducer embedded underneath the microstructure. As shown in Figure 1a, a tactile sensor is held by fingers above an artery and is pressed against the artery with contact pressure *P_c_*, to establish the tissue–sensor contact so that the true pulse signal in an artery: arterial wall displacement *y*(*t*) can go through the TCS stack and cause deflection in the microstructure, which is recorded as the measured pulse signal *x*(*t*). Due to MA, the sensor substrate encounters a time-varying displacement *z_b_*(*t*). By considering *y*(*t*) as the true pulse signal, instead of pulsatile pressure Δ*p*(*t*), the influence of pulse measurement on the true pulse signal in an artery is neglected [7,8].

A PPG sensor contains a light emitter and a photodetector (PD), detects blood volume change in an artery, and does not need a deformable microstructure [23]. As shown in Figure 1b, tape is used to fix a PPG sensor with *P_c_* for establishing the tissue–sensor contact. The light sent by the light emitter is partially absorbed by blood in the artery, and the reflected light goes through the TCS stack and is detected by the PD as the measured pulse signal. Due to MA, the PPG sensor itself encounters a time-varying displacement *x_b_*(*t*).

### 2.2. An SDOF Model of MA in a Measured Pulse Signal

**Figure 2:**
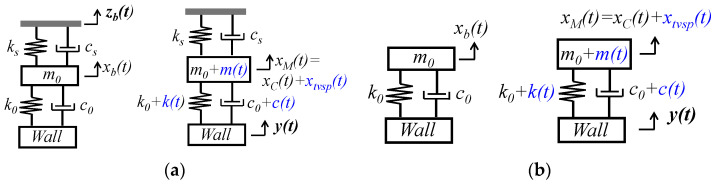
An SDOF model of MA in which the TCS stack is modeled as an SDOF system with arterial wall displacement *y*(*t*) as base excitation in pulse measurement using (**a**) a tactile sensor with MA *z_b_*(*t*) at the sensor substrate and (**b**) a PPG sensor with MA *x_b_*(*t*) at the mass.

The TCS stack between an artery and a sensor, shown in Figure 1, can be treated as an SDOF system to capture its dynamic behavior involved in pulse measurement. As shown in Figure 2a, due to its deformability, the tissue forms an SDOF system with spring stiffness *k*_0_, damping coefficient *c*_0_, and equivalent mass *m*_0_. Note that *P_c_* presets the nominal values of *m*_0_, *k*_0_, and *c*_0_ and causes a static displacement in the sensor. This static displacement is not shown here and is excluded from the analysis, since its effect on a measured pulse signal is fully accounted for by the preset nominal values. With its substrate being fixed, the tactile sensor itself can be treated as another SDOF system with its own spring stiffness *k_s_* and damping coefficient *c_s_* from its microstructure. Since the sensor and the TCS stack join their mass together, the TCS stack remains as an SDOF system. Both the sensor and the tissue contribute to *m*_0_. As shown in Figure 2b, a PPG sensor contributes only to the mass *m*_0_ of the SDOF system of the TCS stack [7].

As shown in Figure 2a, an MA causes $z_{b}(t)$ at the sensor substrate:

(1) $$z_{b}(t)=z_{b0}e^{j{(\omega }_{b}t+\varphi_{b})}$$

where $z_{b0}$, $\varphi_{b}$, and $\omega_{b}$ are the amplitude, phase, and angular frequency of $z_{b}(t)$, respectively. This displacement serves as the base excitation for the SDOF system and leads to the displacement $x_{b}(t)$ at the mass [8]:

(2) $$m_{0}\frac{d^{2}x_{b}\left(t\right)}{{dt}^{2}}+\left(c_{0}+c_{s}\right)\frac{dx_{b}\left(t\right)}{dt}+\left(k_{0}+k_{s}\right)\cdot x_{b}\left(t\right)=k_{s}z_{b}\left(t\right)+c_{s}\frac{dz_{b}\left(t\right)}{dt}$$

The sensor measures the relative distance of its substrate to the mass. Thus, the measured baseline drift by the sensor is $x_{b}(t)-z_{b}(t)$. It is $x_{b}(t)$ that causes the time-varying system parameters (TVSP) *m*(*t*), *k*(*t*), and *c*(*t*) of the TCS stack:

(3) $$m=m_{0}+m(t),\;c=c_{0}+c(t),\;k=k_{0}+k\left(t\right)\mathrm{with}\;m\left(t\right),\;k(t),\;c(t)\propto x_{b}(t)$$

The true pulse signal y(t) also serves as the base excitation for the SDOF system and is time harmonic [14,15,16]:

(4) $$y\left(t\right)=y_{0}e^{j{(\omega }_{y}t+\varphi_{y})}$$

where $y_{0}$, $\varphi_{y}$, and $\omega_{y}$ are the amplitude, phase, and angular frequency of y(t), respectively. As shown in Figure 2a, y(t) causes displacement $x_{M}(t)$ at the mass [8]:

(5) $$\left[m_{0}+m\left(t\right)\right]\cdot \frac{d^{2}x_{M}\left(t\right)}{{dt}^{2}}+\left[c_{0}+c\left(t\right)+c_{s}\right]\cdot \frac{dx_{M}\left(t\right)}{dt}+\left[k_{0}+k\left(t\right)+k_{s}\right]\cdot x_{M}\left(t\right)=\left[k_{0}+k\left(t\right)\right]\cdot y\left(t\right)+\left[c_{0}+c(t)\right]\cdot \frac{dy\left(t\right)}{dt}$$

Due to TVSP in Equation (5), $x_{M}\left(t\right)$ takes the following form [8]:

(6) $${x_{M}(t)=x}_{T}(t)e^{j\varphi_{T}\left(t\right)}\;\mathrm{with}\;\omega_{T}(t)=\frac{{d\varphi }_{T}(t)}{dt}$$

where $x_{T}$, $\varphi_{T}$ and $\omega_{T}$ are the instant amplitude, phase, and frequency of $x_{M}(t)$, respectively. The measured pulse signal by a tactile sensor $x_{tactile}\left(t\right)$ becomes

(7) $$x_{tactile}\left(t\right)=x_{M}\left(t\right)+x_{b}(t)-z_{b}(t)\mathrm{with}\;x_{M}\left(t\right)=x_{C}\left(t\right)+x_{tvsp}(t)$$

where $x_{b}(t)-z_{b}(t)$ is the baseline drift, $x_{C}\left(t\right)$ is the measured pulse signal when free of MA (i.e., free of TVSP), and $x_{tvsp}(t)$ is the TVSP-generated distortion in Equation (5).

When free of MA, based on Equation (5), the measured pulse signal $x_{C}\left(t\right)\;$ is

(8) $$x_{C}\left(t\right)=x_{0}e^{j{(\omega }_{x}t+\varphi_{x})}=G_{0}e^{j\varphi_{0}}y_{0}e^{j{(\omega }_{y}t+\varphi_{y})}$$

(9) $$\mathrm{with}\;G_{0}e^{j\varphi_{0}}\;=\frac{k_{0}+c_{0}j\omega_{y}}{\left[{-m}_{0}\omega_{y}^{2}+\left(c_{0}+c_{s}\right)j\omega_{y}+k_{0}+k_{s}\right]}\;\omega_{x}=\omega_{y};\;\varphi_{x}=\varphi_{y}+\varphi_{0};\;x_{0}=G_{0}y_{0}$$

Thus, the total distortion caused by MA in a measured pulse signal is

(10) $$x_{tactile-MA}\left(t\right)=x_{tvsp}\left(t\right)+x_{b}(t)-z_{b}(t)\mathrm{with}\;x_{tvsp}\left(t\right)=x_{M}\left(t\right)-x_{C}\left(t\right)$$

As shown in Figure 2b, the difference in the SDOF model of MA between a tactile sensor and a PPG sensor is the existence of *k_s_* and *c_s_*. Assigning *k_s_* = *c_s_* = 0 in Equations (2), (5), (8), and (9) gives rise to a PPG signal (i.e., measured pulse signal). A PPG sensor detects the blood volume in an artery [23] and thus a PPG signal is *y*(*t*) when free of MA. However, the length change in the TCS stack due to MA affects optical transduction in a PPG sensor. Optical transduction is extremely complex [23]. For simplicity, this length change is assumed to be recorded at the same scale as *y*(*t*), and then, the PPG signal is [7]

(11) $$\;x_{PPG}\left(t\right)=y\left(t\right)+x_{tvsp}\left(t\right)+x_{b}\left(t\right)$$

The distortion caused by MA in a PPG signal becomes

(12) $$x_{PPG-MA}\left(t\right)=x_{tvsp}\left(t\right)+x_{b}\left(t\right)$$

Since optical transduction is simplified, Equations (11) and (12) are applicable to both reflection-mode and transmission-mode PPG sensors.

### 2.3. A Generalized SDOF-Model-Based Time–Frequency (SDOF-TF) Method

#### 2.3.1. Nature of an Arterial Pulse Signal

An arterial pulse signal *y*(*t*) is a collection of multiple harmonics of HR [16,46]:

(13) $$y\left(t\right)=\sum_{i=1}^{N}A_{i}\cdot cos(2\pi {if}_{C}t+\phi_{0}+\psi \left(t\right))\mathrm{with}\;\psi (t)=R\cdot \sin\left(2\pi f_{r}t+\alpha_{0}\right)$$

where *f_C_* is the frequency of constant HR; *A_i_* and *ϕ*_0_ are the amplitude and initial phase of the *i*th harmonic, respectively; and *ψ*(*t*) is related to respiration, with *R*, *f_r_*, and *α*_0_ being its amplitude, frequency, and initial phase, respectively. Note that *A_i_*, *f_C_*, and *ϕ*_0_ are all constant, and the effect of PF on HR is excluded in Equation (13). The frequency of the *i*th harmonic is altered by *ψ*(*t*): *i*·*f_C_* − *R*·*f_r_*·cos(2π*f_r_t* + *α*_0_). As such, the respiration signal *r*(*t*) embedded in the frequency of the *i*th harmonic is

(14) $$r\left(t\right)=B\cos\left(2\pi f_{r}t+\alpha_{0}\right)\mathrm{with}\;B=-R{\cdot f}_{r}$$

where *B* is respiration modulation, indicating the strength of RSA on altering HR [46].

#### 2.3.2. A Generalized SDOF-TF Method

**Figure 3:**
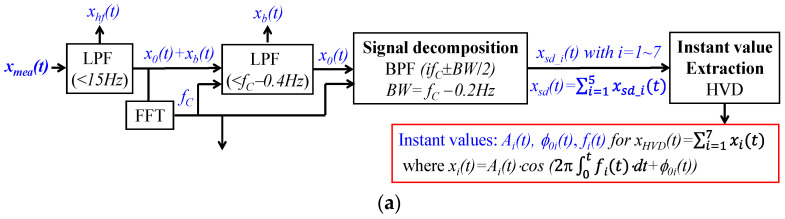

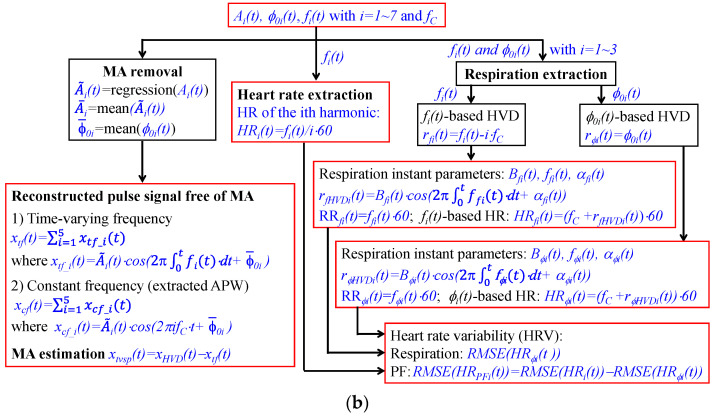
The block diagram of a generalized SDOF-TF method for MA removal and reconstruction of a measured pulse signal free of MA and extraction of APW, HR, and respiration parameters: (**a**) extraction of the instant parameters of each harmonic and (**b**) post-processing of each harmonic.

Figure 3 depicts the generalized SDOF-TF method for MA removal. As shown in Figure 3a, a measured pulse signal *x_mea_*(*t*) at rest contains baseline drift *x_b_*(*t*) (<0.7 Hz) and high-frequency noise *x_hf_*(*t*) (>15 Hz) and *x*_0_(*t*) corresponding to the true pulse signal:

(15) $$x_{mea}\left(t\right)=x_{0}\left(t\right)+x_{b}\left(t\right)+x_{hf}\left(t\right)$$

First, a low-pass filter (LPF) with a cutoff frequency of 15 Hz is used to remove *x_hf_*(*t*). Then, a fast Fourier transform (FFT) is conducted on the signal to obtain frequency *f_C_* from the 1st harmonic. An LPF with a cutoff frequency of *f_C_* − 0.4 Hz is used to remove *x_b_(t).* A bandpass filter (BPF) with cutoff frequencies of *i*·*f_C_* ± *BW*/2, with *BW* = *f_C_* − 0.2 Hz, is used to separate the *i*th harmonic *x_sd_i_*(*t*) from *x*_0_(*t*):

(16) $$\;x_{sd}(t)=\sum_{i=1}^{N}x_{sd\_i}\left(t\right)$$

Finally, HVD [21] is used to calculate instant (time-varying) parameters of each harmonic:

(17) $$x_{HVD}\left(t\right)=\sum_{i=1}^{N}x_{i}\left(t\right)=\sum_{i=1}^{N}A_{i}\left(t\right)\cdot cos(2\pi \cdot \int f_{i}\left(t\right)dt+\phi_{0i}(t))$$

where *A_i_*(*t*), *f_i_*(*t*), and *ϕ*_0*i*_(*t*) denote the instant amplitude, frequency, and initial phase of the *i*th harmonic, respectively. As compared to *x_sd_*(*t*), broadband noise is greatly alleviated in *x_HVD_*(*t*) [21].

As shown in Figure 3b, to remove MA, the regression line of *A_i_*(*t*): ${\tilde{A}}_{i}\left(t\right)$ and mean of *ϕ*_0*i*_(*t*): ${\bar{\phi }}_{0i}$ are obtained. Then, the pulse signal free of MA can be reconstructed with time-varying frequency *f_i_*(*t*):

(18) $$x_{tf}\left(t\right)=\sum_{i=1}^{5}x_{tf\_i}\left(t\right)\;with\;x_{tf\_i}\left(t\right)={\tilde{A}}_{i}\left(t\right)\cdot cos(2\pi \cdot \int f_{i}\left(t\right)dt+{\bar{\phi }}_{0i})$$

Accordingly, *x_TVSP_*(*t*) can be calculated as

(19) $$x_{tvsp}\left(t\right)=x_{HVD}\left(t\right)-x_{tf}\left(t\right)$$

The corresponding pulse signal with constant frequency (i.e., constant HR) is reconstructed to examine the effect of time-varying frequency (or HRV) on the APW of a pulse signal:

(20) $$x_{cf}\left(t\right)=\sum_{i=1}^{5}x_{cf\_i}\left(t\right)\;with\;x_{cf\_i}\left(t\right)={\tilde{A}}_{i}\left(t\right)\cdot cos(2\pi {if}_{C}t+{\bar{\phi }}_{0i})$$

To extract HR from the *i*th harmonic, the following equation is used:

(21) $${HR}_{i}(t)=\;f_{i}\left(t\right)/i\cdot 60(beats\;per\;minute,bpm)$$

Extraction of the respiration signal is conducted on both *f_i_*(*t*) and *ϕ*_0*i*_(*t*). As to *f_i_*(*t*)-based extraction, based on Equation (13), the respiration signal from the *i*th harmonic is

(22) $$\;r_{fi}\left(t\right)=f_{i}\left(t\right)-i\cdot f_{C}$$

HVD is used to extract the instant parameters of *r_fi_*(*t*):

(23) $$r_{fHVDi}\left(t\right)=B_{fi}\left(t\right)\cdot cos(2\pi \int_{0}^{t}f_{fi}\left(t\right)\cdot dt+\alpha_{0fi}(t))$$

where *B_fi_*_0_(*t*), *f_fi_*(*t*), and α_0*fi*_(*t*) denote the instant amplitude, frequency, and initial phase of $r_{fHVDi}\left(t\right)$, respectively. As to *ϕ*_0*i*_(*t*)-based extraction, the respitation signal from the *i*th harmonic is simply *ϕ*_0*i*_(*t*). HVD is used to extract the instant parameters of *ϕ*_0*i*_(*t*):

(24) $$r_{\phi HVDi}\left(t\right)=B_{\phi i}\left(t\right)\cdot cos(2\pi \int_{0}^{t}f_{\phi i}\left(t\right)\cdot dt+\alpha_{0\phi i}(t))$$

where *B_ϕi_*_0_(*t*), *f_ϕi_*(*t*), and *α*_0*ϕi*_(*t*) denote the instant amplitude, frequency, and initial phase of $r_{\phi HVDi}\left(t\right)$, respectively. Consequently, the HR accounting for respiration becomes

(25) $${HR}_{fi}\left(t\right)=(r_{fHVDi}\left(t\right)+f_{C})\cdot 60\;(\mathrm{bpm});\;{HR}_{\phi i}\left(t\right)=(r_{\phi HVDi}\left(t\right)+f_{C})\cdot 60\;(\mathrm{bpm})$$

Here, HRV is calculated using root mean squared error (RMSE). While RMSE(*HR_i_*(*t*)) is the total HRV due to respiration and PF, RMSE(*HR_ϕi_*(*t*)) is the HRV due to respiration. Then, the HRV due to PF is calculated as

(26) $${RSME\left({HR}_{PFi}\left(t\right)\right)=RSME\left({HR}_{i}\left(t\right)\right)-RSME(HR}_{\phi i}\left(t\right))$$

### 2.4. Calculation

#### 2.4.1. The Effects of MA and Respiration on a Measured Pulse Signal

While the true pulse signal *y(t*) is affected by respiration (PF is not considered here and will be considered in Section 3), a measured pulse signal is affected by *y(t*), the TCS stack, and MA. To gain insights into the effects of MA and respiration on a measured pulse signal with clarity, a simplified model is created based on the SDOF model of MA for calculation. In this model, *k_s_* and *c_s_* are assumed to be zero, since their existence only affects the absolute values, and will not alter the nature of the effects of MA and respiration on the frequency spectrum of a measured pulse signal. Because the values of *m*_0_, *k*_0_, and *c*_0_ and the mathematical relations between MA and TVSP are all unknown, they are all assumed such that the generated distortion is noticeable in the frequency domain.

The 3rd harmonic with respiration is used to represent the true pulse signal:

(27) $$y\left(t\right)=1\cdot cos(2\pi {3\cdot f}_{C}t+0.3\sin\left(2\pi 0.2t\right))\;\mathrm{with}\;f_{C}=1.2\mathrm{Hz}$$

Due to respiration, its frequency is 3·*f_C_* − 0.06cos(2π0.2t). Both MA at rest (<0.7 Hz) and MA during activities (>0.7 Hz) are considered. The extracted *x_b_*(*t*) from a measured pulse signal pre-exercise using a tactile sensor and from a PPG signal are used as the MA at rest. The MA during activities is assumed to be

(28) $$x_{b}(t)=exp(-0.01t)\cdot cos(2\pi ft)\;\mathrm{with}\;f=3f_{C}\;\mathrm{and}\;f=3f_{C}-0.3\mathrm{Hz}$$

First, we calculate the measured pulse signal with/without a pre-defined MA and with/without respiration in the time domain to obtain *x_C_*(*t*), *x_M_*(*t*), and *x_tvsp_*(*t*). Afterward, an FFT is conducted on them to examine the effects of MA and respiration on their frequency spectrum. Finally, HVD is conducted on the measured pulse signal *x_M_*(*t*) = *x_C_*(*t*) + *x_tvsp_*(*t*) at rest and *x_M_*(*t*) + *x_b_*(*t*) during activities to examine the effects of MA and respiration on its instant parameters. The details about the calculation can be found in the literature [7,8]. All the calculations are conducted in MATLAB2025a.

#### 2.4.2. Application of the Generalized SDOF-TF Method to Measured Pulse Signals

Based on the block diagram in Figure 3, the algorithm for the SDOF-TF method is written in MATLAB2025a. Three pulse signals were measured at the wrist using a tactile sensor from one healthy subject at rest under three conditions: pre-exercise, 1 min post-exercise, and 5 min post-exercise (approved by the Institutional Review Board (IRB) at Old Dominion University), and were analyzed here. Additionally, four PPG signals from four subjects under four physiological conditions: non-AF, AF, critically ill, and during activities, from different datasets [25], were also analyzed. The non-AF and AF subjects in the dataset MIMIC PERform AF were measured at the finger at rest in the same study. The critically ill subjects in the dataset BIDMC were measured at the finger at rest in another study. The subjects in the dataset PPG DaLiA were measured at the wrist during activities. The details about these datasets can be found in the literature [9]. To avoid the suspicion of selecting PPG signals to achieve the desired results, it is always the PPG signal from the first subject in each dataset that is selected, from which an 80s long segment with low-level MA is further selected for analysis. It should be emphasized that choosing these seven pulse signals under different physiological conditions using two different types of sensors is aimed at examining the effectiveness and generalizability of the SDOF-TF method for the removal of MA at rest from a measured pulse signal and extraction of its APW, HR, and respiration parameters, rather than achieving statistical significance.

## 3. Results

### 3.1. The Effects of MA and Respiration on a Measured Pulse Signal from the SDOF Model of MA

**Figure 4:**
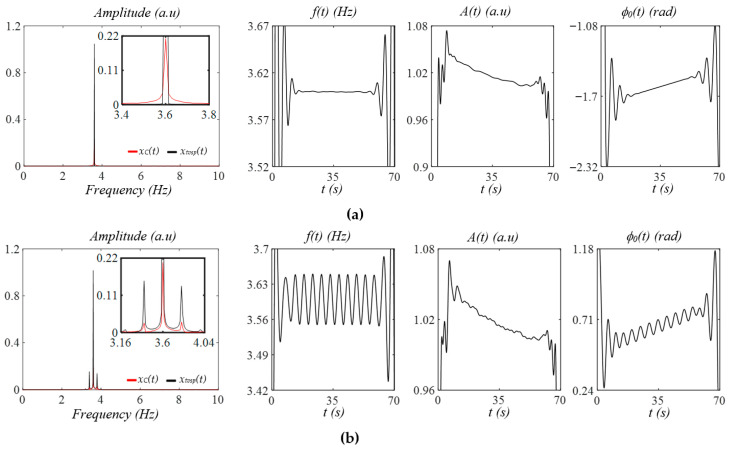
Frequency spectrum of *x_C_*(*t*) and *x_tvsp_*(*t*) and instant parameters *A*(*t*), *f*(*t*), and *ϕ_0_*(*t*), of the measured pulse signal, where *x_M_*(*t*) = *x_C_*(*t*) + *x_tvsp_*(*t*), with the MA at rest, from a tactile sensor (see Figure 8) (**a**) without respiration and (**b**) with respiration.

Figure 4a shows the effects of MA at rest (baseline drift in the measured pulse signal using a tactile sensor in Figure 8) on a measured pulse signal with no respiration. TVSP-generated distortion *x_tvsp_*(*t*) contains sidebands around the signal. While its instant frequency is almost constant and its instant initial phase is a straight line, its instant amplitude is noticeably affected by *x_tvsp_*(*t*). The abrupt changes at the start/end of these instant parameters are the end effects of the Hilbert transform and are meaningless. Figure 4b shows the effect of the same MA at rest with respiration. The measured pulse signal with no *x_b_*(*t*) carries multiple sidebands from respiration, and *x_tvsp_*(*t*) follows the respiration sidebands. The instant frequency and instant initial phase both carry the respiration signal in the HR.

**Figure 5:**
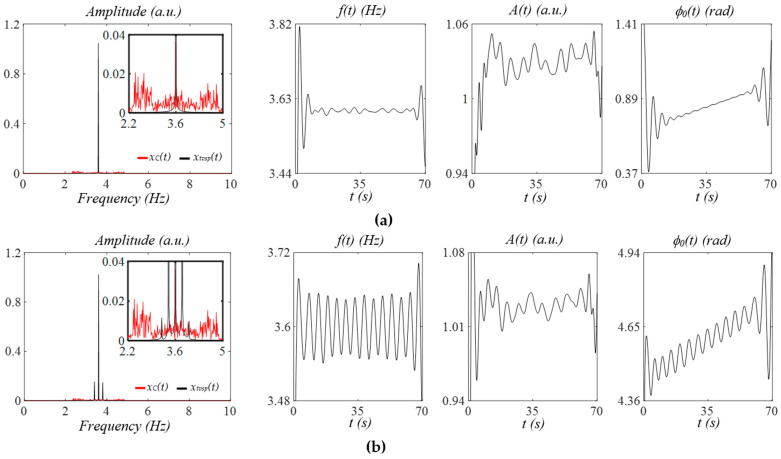
Frequency spectrum of *x_C_*(*t*) and *x_tvsp_*(*t*) and instant parameters *A*(*t*), *f*(*t*), and *ϕ_0_*(*t*) of the measured pulse signal: *x_M_*(*t*) = *x_C_*(*t*) + *x_tvsp_*(*t*), with the MA at rest, from a PPG sensor (see Figure 13) (**a)** without respiration and (**b**) with respiration.

Figure 5a shows the effects of MA at rest (baseline drift in the PPG signal in Figure 13) on a measured pulse signal with no respiration. Similarly, *x_tvsp_*(*t*) contains multiple sidebands around the pulse signal. The instant amplitude of the measured pulse signal is greatly affected by *x_tvsp_*(*t*), and its instant frequency is affected to a very limited extent. In contrast, the instant initial phase is minimally affected by *x_tvsp_*(*t*). Figure 5b shows the effect of the same MA at rest with respiration. The measured pulse signal contains multiple sidebands, and *x_tvsp_*(*t*) follows the respiration sidebands. Both the instant frequency and instant initial phase carry the respiration signal.

**Figure 6:**
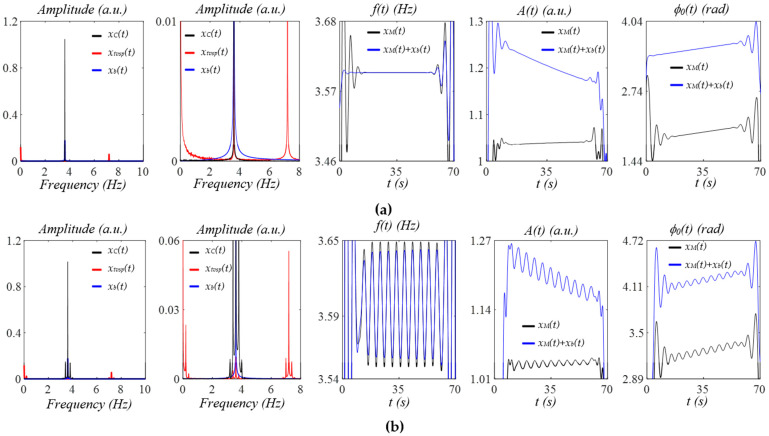
Frequency spectrum of *x_C_*(*t*), *x_b_*(*t*), and *x_tvsp_*(*t*) and instant parameters *A*(*t*), *f*(*t*), and *ϕ_0_*(*t*) of the measured pulse signal: *x_M_*(*t*) + *x_b_*(*t*), and *x_M_*(*t*) = *x_C_*(*t*) + x*_tvsp_*(*t*), with MA during activities at *f* = 3 · *fC* (**a**) without respiration and (**b**) with respiration.

Figure 6a shows the effect of MA: *x_b_*(*t*) = exp(−0.01t)·cos(3·*f_C_*) during activities, whose frequency coincides with that of the pulse signal, with no respiration. Note that *x_tvsp_*(*t*) contains a very small signal coinciding with the harmonic signal and also relatively large signals far off from the pulse signal. Thus, the instant frequency, amplitude, and initial phase of the measured pulse signal *x_M_*(*t*) with no *x_b_*(*t*) are almost unchanged by *x_tvsp_*(*t*). Since *x_b_*(*t*) cannot be filtered out, the measured pulse signal should be *x_M_*(*t*) + *x_b_*(*t*), showing that *x_b_*(*t*) does not affect the instant frequency but causes noticeable changes in the instant amplitude and instant initial phase. Figure 6b shows the effect of the same MA during activities with respiration. Now, *x_tvsp_*(*t*) at different frequencies carries the sidebands from the respiration. The instant frequency, amplitude, and initial phase of *x_M_*(*t*) with no *x_b_*(*t*) all capture the respiration signal. Adding *x_b_*(*t*) to *x_M_*(*t*) does not change the instant frequency but causes a shift in the instant amplitude and instant initial phase. While the instant amplitude is amplified by *x_b_*(*t*), the respiration signal in the instant initial phase remains unchanged.

**Figure 7:**
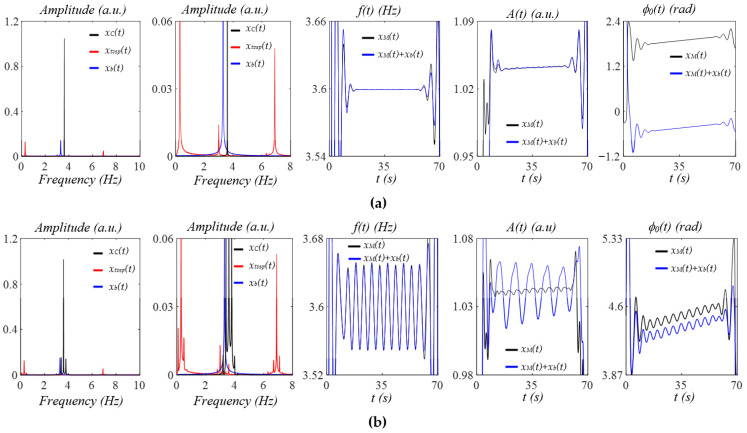
Frequency spectrum of *x_C_*(*t*), *x_b_*(*t*), and *x_tvsp_*(*t*) and instant parameters *A*(*t*), *f*(*t*), and *ϕ_0_*(*t*) of the measured pulse signal: *x_M_*(*t*) + *x_b_*(*t*), and *x_M_*(*t*) = *x_C_*(*t*) + x*_tvsp_*(*t*), with MA during activities at *f* = 3 · *fC* − 0.3 Hz (**a**) without respiration and (**b**) with respiration.

Figure 7a shows the effect of MA: *x_b_(t) =* exp(−0.01t)·cos(3·*f_C_* − 0.3 Hz) during activities, with no respiration. Note that *x_tvsp_*(*t*) contains a small signal, a little off from the harmonic signal, but two relatively large signals far off from the pulse signal. The instant frequency, amplitude, and initial phase of *x_M_*(*t*) are almost unaffected by *x_tvsp_*(*t*). Adding *x_b_*(*t*) to *x_M_*(*t*) causes only a shift in the instant initial phase. Figure 7b shows the effect of the same MA with respiration. The instant frequency, amplitude, and initial phase of *x_M_*(*t*) all carry the respiration signal, but the respiration signal in the instant amplitude is extremely small. Adding *x_b_*(*t*) to *x_M_*(*t*), the measured pulse signal does not cause any changes in the instant frequency, but greatly amplifies the instant amplitude. The respiration signal in the instant initial phase is unaffected. Similar to respiration, PF modulates HR. Thus, HRV, or the time-varying frequency of a measured pulse signal, stems from respiration and PF. Theoretically speaking, the instant frequency and instant initial phase in a measured pulse signal might both carry the respiration signal and the PF information. Yet, the slope of the instant initial phase in the Hilbert transform is not yet well defined. As shown in Figure 4, Figure 5, Figure 6 and Figure 7, this slope varies randomly. Given that PF is not time harmonic, the time-harmonic signal in the instant initial phase is believed to be only from respiration, and then the instant initial phase can be used to extract the respiration signal.

Based on the above results, the key findings about the effects of MA and respiration on the instant parameters of a harmonic in a measured pulse signal are as follows:

1. The MA at rest greatly affects instant amplitude, affects instant frequency to a very limited extent, and has almost no effect on instant initial phase.
2. The MA during activities generates multiple signals at different frequencies. These multiple signals will affect those harmonics in a measured pulse signal whose frequencies are close to theirs.
3. Instant frequency captures HRV (from constant HR or *f_C_*) due to respiration and PF, and instant initial phase captures HRV due to respiration only.
4. Regardless of MA at rest or during activities, instant frequency and instant initial phase carry the respiration signal in HR. The respiration signal in the instant initial phase is almost immune to MA compared to its counterpart in the instant frequency.

### 3.2. Measured Pulse Signals Using a Tactile Sensor

**Figure 8:**
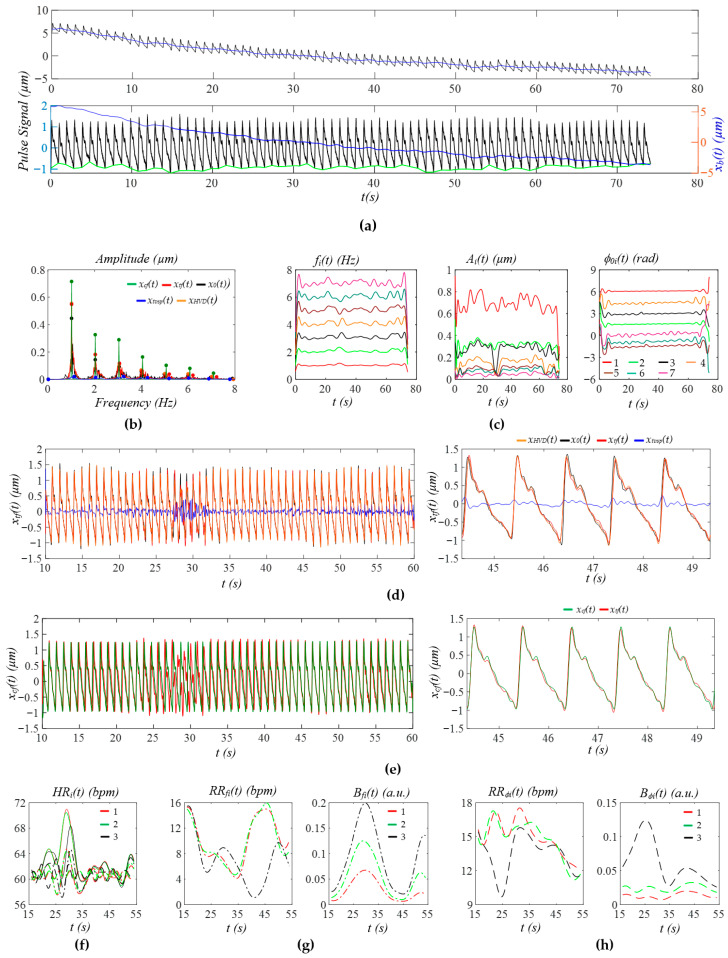
A measured pulse signal of a healthy 28-yr-old male subject pre-exercise: (**a**) original measured pulse signal, *x*_0_(*t*), and *x_b_*(*t*); (**b**) frequency spectrum of *x*_0_(*t*), *x_HVD_*(*t*), *x_tf_*(*t*), *x_cf_*(*t*), and *x_tvsp_*(*t*); (**c**) instant parameters of the harmonics; (**d**) reconstructed pulse signal *x_tf_*(*t*) with *f_i_*(*t*); (**e**) reconstructed pulse signal *x_cf_*(*t*) with *f_C_*; (**f**) HR: *HR_i_*(*t*), *HR_fi_*(*t*), and *HR_ϕi_*(*t*); (**g**) *f_i_*(*t*)-based respiration parameters; and (**h**) *ϕ*_0*i*_(*t*)-based respiration parameters.

As shown in Figure 8a, after the removal of baseline drift *x_b_*(*t*), the measured pulse signal *x*_0_(*t*) does not contain a flat baseline, due to *x_tvsp_*(*t*) and HRV (time-varying frequency). As shown in Figure 8b, *x*_0_(*t*) contains sidebands due to MA, HRV, and broadband noise. While *x_HVD_*(*t*) contains sidebands from MA and HRV, *x_tf_*(*t*) contains sidebands only from HRV, and *x_cf_*(*t*) contains no sidebands due to a constant HR. The calculated *x_tvsp_*(*t*) is small, relative to *x*_0_(*t*). Figure 8c shows the instant parameters of the first seven harmonics of *x*_0_(*t*). Based on the instant amplitude *A_i_*(*t*), the third and fourth harmonics contain some abnormalities compared to the rest of the harmonics. The instant initial phases *ϕ*_0*i*_(*t*) of the first three harmonics are smoother than their counterparts of the higher harmonics, possibly due to their low signal-to-noise ratio. Figure 8d compares the reconstructed pulse signal with time-varying frequency *x_tf_*(*t*), and with *x_HVD_*(*t*), *x*_0_(*t*), and *x_tvsp_*(*t*). Compared with *x*_0_(*t*), broadband noise in *x_HVD_*(*t*) is greatly reduced. Compared to *x_HVD_*(*t*), *x_tf_*(*t*) removes MA (i.e., *x_tvsp_*(*t*)) from it. The large *x_tvsp_*(*t*) around 25–32 s is from a sudden reduction in HR and amplitude of the pulse signal in Figure 8a.

Figure 8e compares *x_tf_*(*t*) with the reconstructed pulse signal with constant frequency *x_cf_*(*t*). Time-varying frequency lowers the dicrotic notch. Figure 8f compares the *HR_i_*(*t*) (accounting for respiration and PF) with *HR_fi_*(*t*) and *HR_ϕi_*(*t*) (accounting solely for respiration; see Equation (23)). Here, only the first three harmonics are used, because the higher harmonics are believed to carry a low signal-to-noise ratio. The difference between *HR_i_*(*t*) and *HR_fi_*(*t*) or *HR_ϕi_*(*t*) represents the effect of PF on HR. While Figure 8g illustrates instant RR: *RR_fi_*(*t*), and instant respiration modulation: *B_fi_*(*t*), from *f_i_*(*t*), Figure 8h illustrates instant RR: *RR_ϕi_*(*t*) and instant respiration modulation: *B_ϕi_*(*t*), from *ϕ_i_*(*t*). The difference between them implies that the respiration signals in *f_i_*(*t*) and *ϕ_i_*(*t*) are different.

**Figure 9:**
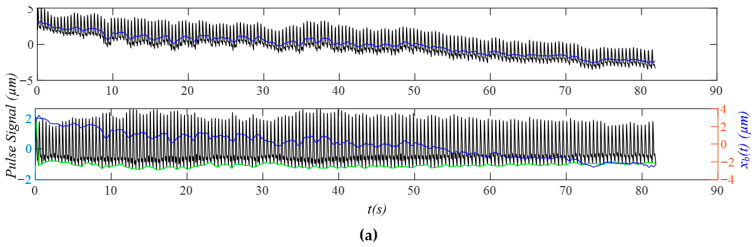

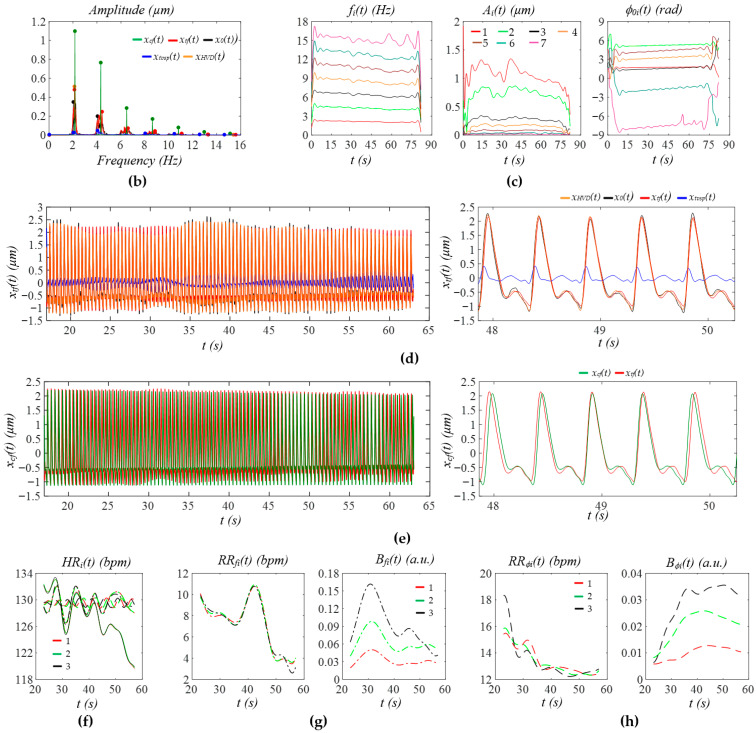
A measured pulse signal of a healthy 28-yr-old male subject 1 min post-exercise: (**a**) original measured pulse signal, *x*_0_(*t*), and *x_b_*(*t*); (**b**) frequency spectrum of *x*_0_(*t*), *x_HVD_*(*t*), *x_tf_*(*t*), *x_cf_*(*t*), and *x_tvsp_*(*t*); (**c**) instant parameters of the harmonics; (**d**) reconstructed pulse signal *x_tf_*(*t*) with *f_i_*(*t*); (**e**) reconstructed pulse signal *x_cf_*(*t*) with *f_C_*; (**f**) HR: *HR_i_*(*t*), *HR_fi_*(*t*), and *HR_ϕi_*(*t*); (**g**) *f_i_*(*t*)-based respiration parameters; and (**h**) *ϕ*_0*i*_(*t*)-based respiration parameters.

Figure 9a shows the measured pulse signal 1 min post-exercise of the same subject in Figure 8. In Figure 9b, the wide sidebands of *x_tf_*(*t*) imply a large HRV. Figure 9c does not show abnormalities in instant amplitude. In Figure 9d, the small difference between *x_HVD_*(*t*) and *x*_0_(*t*) shows low broadband noise, possibly due to the large amplitude of the pulse signal. The small difference between *x_HVD_*(*t*) and *x_tf_*(*t*) indicates a small *x_tvsp_*(*t*). As shown in Figure 9e, although HRV is high for the whole pulse signal, HRV (or time-varying frequency) between consecutive pulse cycles is low. Thus, no noticeable difference between *x_tf_*(*t*) and *x_cf_*(*t*) is observed in terms of APW. The dicrotic notch in Figure 9e is much lower than that in Figure 8e. As shown in Figure 9f, *HR_i_*(*t*) shows a large decreasing trend with time, while *HR_fi_*(*t*) and *HR_ϕi_*(*t*) do not capture such a trend. Meanwhile, *HR_ϕi_*(*t*) is noticeably smaller than *HR_i_(t).* In Figure 9g,h, instant RR captures such a decreasing trend, but not instant respiration modulation.

**Figure 10:**
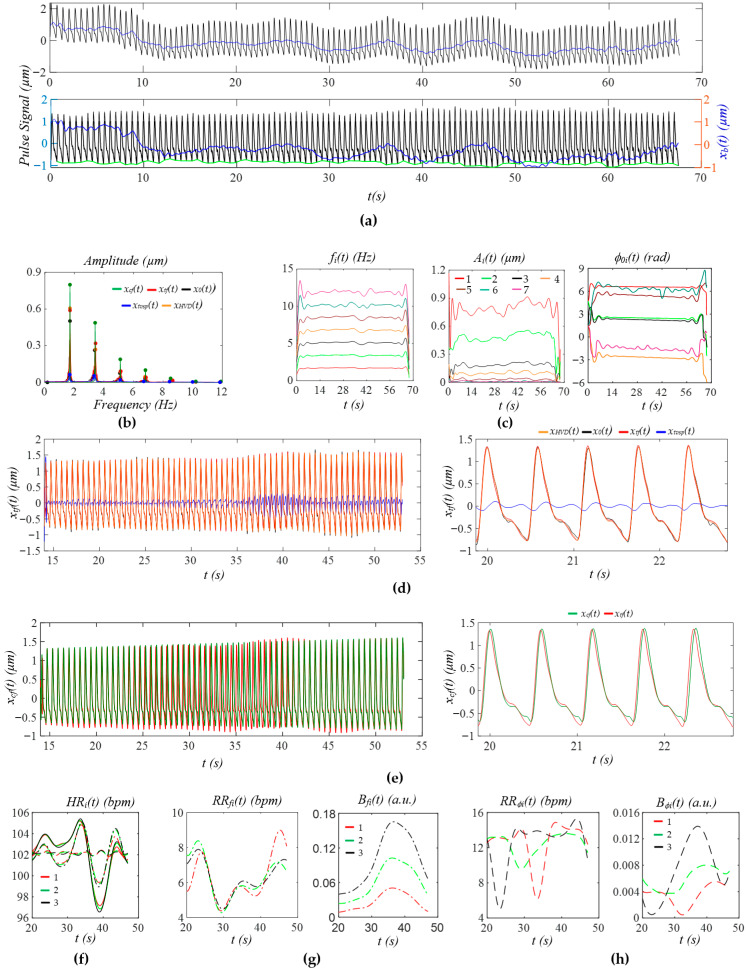
A measured pulse signal of a healthy 28-yr-old male subject 5 min post-exercise: (**a**) original measured pulse signal, *x*_0_(*t*), and *x_b_*(*t*); (**b**) frequency spectrum of *x*_0_(*t*), *x_HVD_*(*t*), *x_tf_*(*t*), *x_cf_*(*t*), and *x_tvsp_*(*t*); (**c**) instant parameters of the harmonics; (**d**) reconstructed pulse signal *x_tf_*(*t*) with *f_i_*(*t*); (**e**) reconstructed pulse signal *x_cf_*(*t*) with *f_C_*; (**f**) HR: *HR_i_*(*t*), *HR_fi_*(*t*), and *HR_ϕi_*(*t*); (**g**) *f_i_*(*t*)-based respiration parameters; (**h**) and *ϕ*_0*i*_(*t*)-based respiration parameters.

Figure 10a shows the measured pulse signal 5 min post-exercise of the same subject in Figure 8. As compared to Figure 9b, the sidebands of *x_tf_*(*t*) in Figure 10b are much narrower, indicating a smaller HRV than pre-exercise. Figure 9c does not show any abnormality in instant amplitude. As shown in Figure 9d, broadband noise is low, and *x_tvsp_*(*t*) is small. The dicrotic notch in Figure 10d is at a similar location but is much shallower, as compared to Figure 9d. As shown in Figure 10e, and as compared to *x_cf_*(*t*), time-varying frequency alters the shape of the dicrotic notch a bit and removes the tiny peak toward the end of a pulse cycle. As shown in Figure 10f, *HR_i_*(*t*) does not decrease with time, and *HR_ϕi_*(*t*) is extremely small, as compared to *HR_fi_(t).* In Figure 10g,h, instant RR does not show any decreasing trend either.

**Figure 11:**
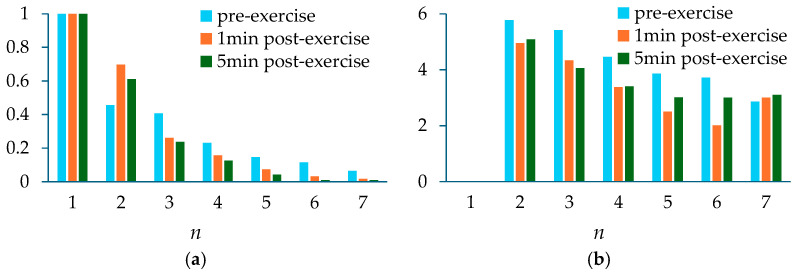
Comparison of *A_cf_i_*/*A_cf__*_1_ and $\bar{\phi }$_0*i*_ − $\bar{\phi }$_01_ of the harmonics of the measured pulse signals between pre-exercise and 1 min and 5 min post-exercise: (**a**) *A_cf_i_*/*A_cf__*_1_; (**b**) $\bar{\phi }$_0*i*_ − $\bar{\phi }$_01_.

Contact pressure and individual variations (e.g., thin tissue versus thick tissue) greatly affect the amplitude of a measured pulse signal. To alleviate their effect, Table 1 compares the normalized amplitudes and initial phase differences in the harmonics relative to the first harmonic between three physiological conditions. Note that *A_cf_i_*/*A_cf__*_1_ is from the extracted APW. Here, *x_cf_*(*t*) is treated as extracted APW, because (1) it is free of HRV and eliminates APW variation between pulse cycles, and (2) HRV varies between physiological conditions. Although $\bar{A}$*_i_*/$\bar{A}$_1_ is from the HVD analysis of *x_i_*(*t*), it is almost identical to *A_cf_i_*/*A_cf__*_1_, indicating the effectiveness of using the regression line to remove *x_tvsp_*(*t*). Similarly, $\bar{\phi }$_0*i*_ is from the extracted APW. In contrast, *A_x_*_0*i*_/*A_x_*_01_ from *x*_0_(*t*) contains broadband noise, HRV, and *x_tvsp_*(*t*), and *A_tf_i_*/*A_tf__*_1_ contains HRV, yielding different values. Thus, *A_cf_i_*/*A_cf__*_1_ and $\bar{\phi }$_0*i*_ − $\bar{\phi }$_01_ are chosen for comparison. As shown in Figure 11, compared to pre-exercise, only the normalized amplitude of the second harmonic is increased, and the normalized amplitudes of the other harmonics are all lowered 1 min and 5 min post-exercise. As compared to pre-exercise, the initial phase differences in the second~sixth harmonics are all moved ahead.

Table 2 compares the calculated results on HR and respiration parameters. As compared to pre-exercise, HR, or mean(*HR_i_*(*t*)), is significantly increased 1 min post-exercise and goes down 5 min post-exercise. The total HRV (respiration and PF), calculated as RMSE(*HR_i_*(*t*)), is reduced 5 min post-exercise, as compared to pre-exercise. The large HRV 1 min post-exercise is due to the decreasing trend in HR over time.

The *f_i_*(*t*)-based RR, or mean(*RR_fi_*(*t*)), is below the normal RR range (12~20 Hz) [16]. Thus, the parameters associated with it are listed but are not used for comparison. The *ϕ_i_*(*t*)-based RR, mean(*RR_ϕi_*(*t*)), falls into the normal RR range. In the *ϕ_i_*(*t*)-based respiration modulation, mean(*B_ϕi_*(*t*)) is below 0.1 for the three harmonics under the three conditions. Note that mean(*B_ϕi_*(*t*)) is an indicator of HRV due to respiration, or RMSE(*HR_ϕi_*(*t*)).

While mean(*HR_ϕi_*(*t*)) is the HR accounting for respiration, RMSE(*HR_ϕi_*(*t*)) is the HRV due to respiration. Although RMSE(*HR_i_*(*t*)) 5 min post-exercise is lower than pre-exercise, RSME(*HR_ϕi_*(*t*)) 5 min post-exercise is almost four times lower than pre-exercise, indicating a significant reduction effect of exercise on HRV due to respiration. RMSE(*HR_PFi_*(*t*)) is the HRV due to PF, which is reduced by 20% 5 min post-exercise, as compared to pre-exercise. HRV due to respiration occupies 25% of the total HRV (or RMSE(*HR_i_*(*t*)) pre-exercise, but HRV due to respiration occupies only 10% of the total HRV 1 min and 5 min post-exercise.

### 3.3. PPG Signals

**Figure 12:**
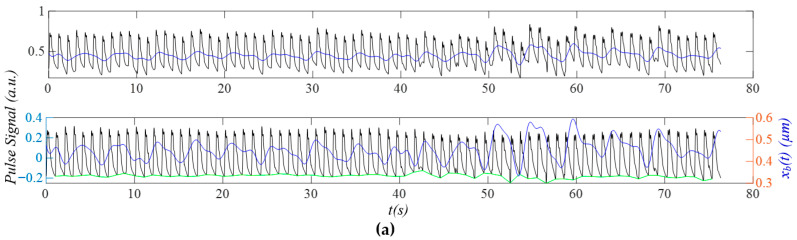

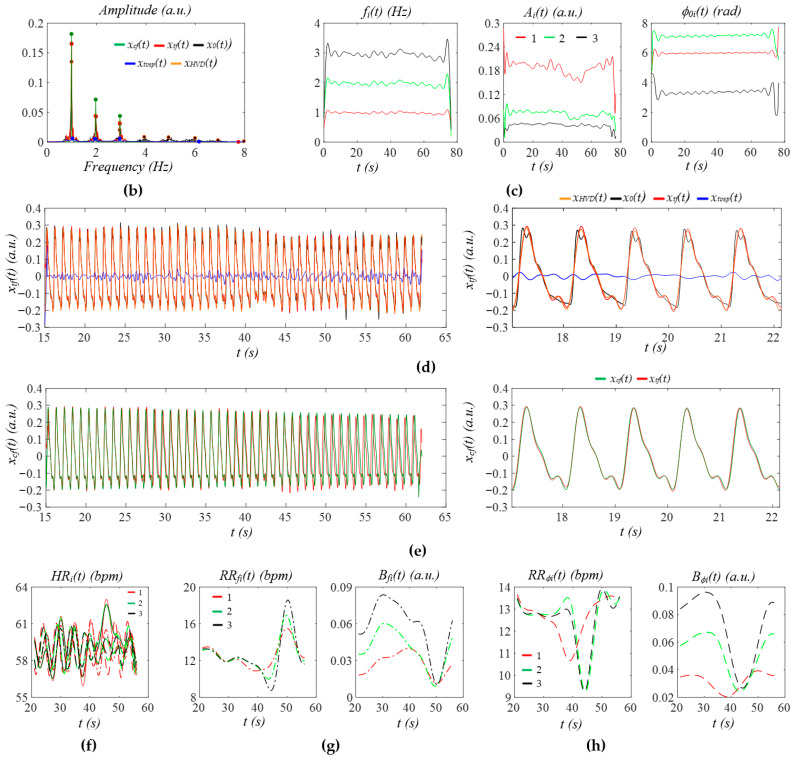
The PPG signal (segment: 70~150 s) of the first non-AF subject (MIMIC PERform AF Dataset): (**a**) original measured pulse signal, *x*_0_(*t*), and *x_b_*(*t*); (**b**) frequency spectrum of *x*_0_(*t*), *x_HVD_*(*t*), *x_tf_*(*t*), *x_cf_*(*t*), and *x_tvsp_*(*t*); (**c**) instant parameters of the harmonics; (**d**) reconstructed pulse signal *x_tf_*(*t*) with *f_i_*(*t*); (**e**) reconstructed pulse signal *x_cf_*(*t*) with *f_C_*; (**f**) HR: *HR_i_*(*t*), *HR_fi_*(*t*), and *HR_ϕi_*(*t*); (**g**) *f_i_*(*t*)-based respiration parameters; and (**h**) *ϕ*_0*i*_(*t*)-based respiration parameters.

**Figure 13:**
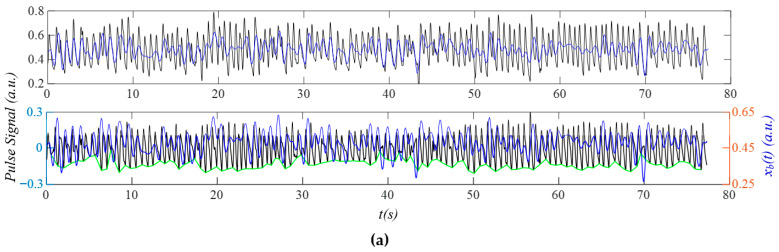

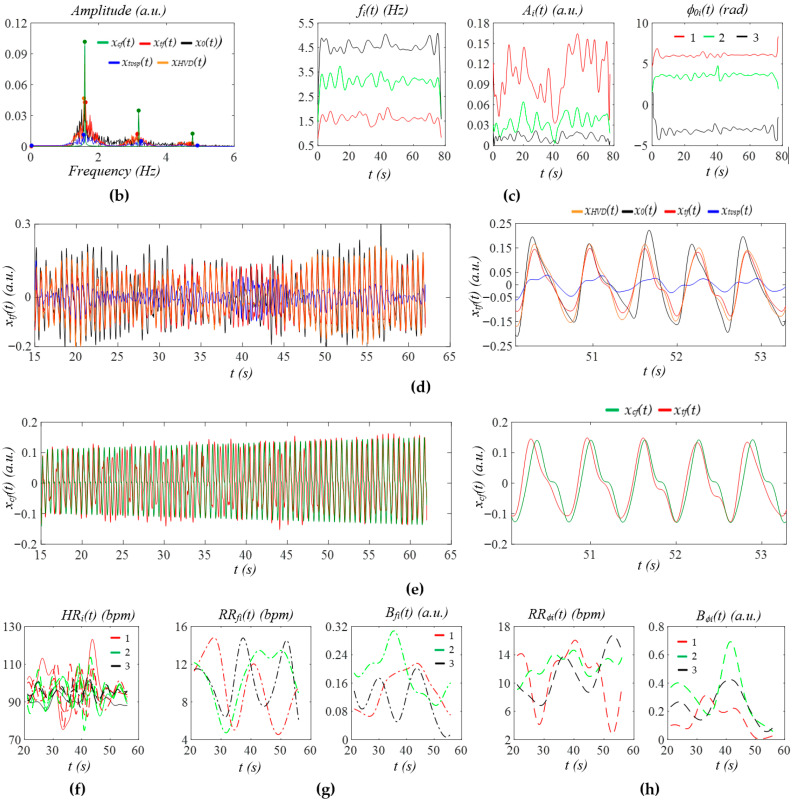
The PPG signal (segment: 70~150 s) of the first AF subject (MIMIC PERform AF dataset): (**a**) original measured pulse signal, *x*_0_(*t*), and *x_b_*(*t*); (**b**) frequency spectrum of *x*_0_(*t*), *x_HVD_*(*t*), *x_tf_*(*t*), *x_cf_*(*t*), and *x_tvsp_*(*t*); (**c**) instant parameters of the harmonics; (**d**) reconstructed pulse signal *x_tf_*(*t*) with *f_i_*(*t*); (**e**) reconstructed pulse signal *x_cf_*(*t*) with *f_C_*; (**f**) HR: *HR_i_*(*t*), *HR_fi_*(*t*), and *HR_ϕi_*(*t*); (**g**) *f_i_*(*t*)-based respiration parameters; and (**h**) *ϕ*_0*i*_(*t*)-based respiration parameters.

Figure 12a shows the PPG signal (segment: 70~150 s) of the first non-AF subject in the dataset MIMIC PERform AF. After the removal of *x_b_*(*t*), all the pulse cycles are brought to a similar level, except for the pulse cycles near 50 s, which experience larger MA. Figure 12b shows the FFT analysis of *x*_0_(*t*), *x_tf_*(*t*), *x_HVD_*(*t*), *x_cf_*(*t*), and *x_tvsp_*(*t*), revealing small sidebands from HRV and a small *x_tvsp_*(*t*). Here, only the first three harmonics are analyzed for comparison with the PPG signal from an AF subject (see Figure 13). In Figure 12c, large instant amplitude changes around 50 s correspond to larger MA in the PPG signal at the time. The respiration signal in instant initial phase is more noticeable, as compared to the measured pulse signals using a tactile sensor.

Figure 12d compares the reconstructed pulse signal with time-varying frequency *x_tf_*(*t*) with *x_HVD_*(*t*), *x*_0_(*t*), and *x_tvsp_*(*t*). The difference between *x*_0_(*t*) and *x_HVD_*(*t*) indicates broadband noise, which causes a large difference at the top and bottom of the pulse cycles. It is interesting to note that broadband noise causes a fake notch near the top and misses a notch near the bottom of a pulse cycle. The small difference between *x_tf_*(*t*) and *x_HVD_*(*t*) indicates a small MA (or small *x_tvsp_*(*t*)). As shown in Figure 12e, due to low HRV, the difference between *x_cf_*(*t*) and *x_tf_*(*t*) is very small. Figure 12f shows that *HR_i_*(*t*), *HR_fi_*(*t*), and *HR_ϕi_*(*t*) are comparable in amplitude, possibly indicating that respiration affects HRV to a much larger extent than PF. As shown in Figure 12g,h, instant RR and instant respiration modulation from *f_i_*(*t*) and *ϕ_i_*(*t*) are different.

Figure 13a shows the PPG signal (segment: 70~150 s) from the first AF subject in the dataset: MIMIC PERform AF. As compared to Figure 12a, *x_b_*(*t*) varies dramatically with time, and a large variation exists between pulse cycles. As shown in Figure 13b, the large and wide sidebands of *x_tf_*(*t*) indicate large HRV, and the wide and relatively large sidebands of *x_tvsp_*(*t*) indicate large MA, as compared to Figure 12b. Only the first three harmonics are analyzed, since the fourth harmonic is extremely small. Similarly to Figure 12c, the instant initial phase noticeably captures the respiration signal in Figure 13c. Furthermore, the large MA around 38~45 s is captured by the instant amplitude.

As shown in Figure 13d, the large difference between *x_HVD_*(*t*) and *x*_0_(*t*) indicates large broadband noise, which greatly distorts a pulse cycle. The moderate difference between *x_tf_*(*t*) and *x_HVD_*(*t*) indicates a relatively large *x_tvsp_*(*t*). As shown in Figure 13e, due to the large change in time-varying frequency (large HRV), the difference between *x_cf_*(*t*) and *x_tf_*(*t*) is remarkable, in the sense that the dicrotic notch is moved up and down by HRV. Figure 13f shows that *HR_i_*(*t*), *HR_fi_*(*t*), and *HR_ϕi_*(*t*) are comparable in amplitude, possibly indicating that respiration affects HRV more dramatically than PF. Figure 13g,h show that the instant RR and instant respiration modulation from *f_i_*(*t*) and *ϕ_i_*(*t*) are different.

**Figure 14:**
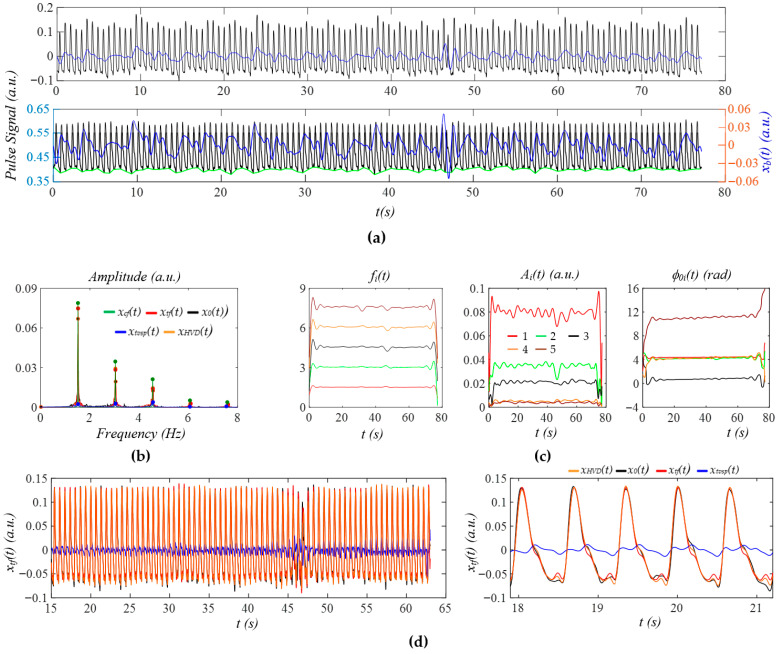

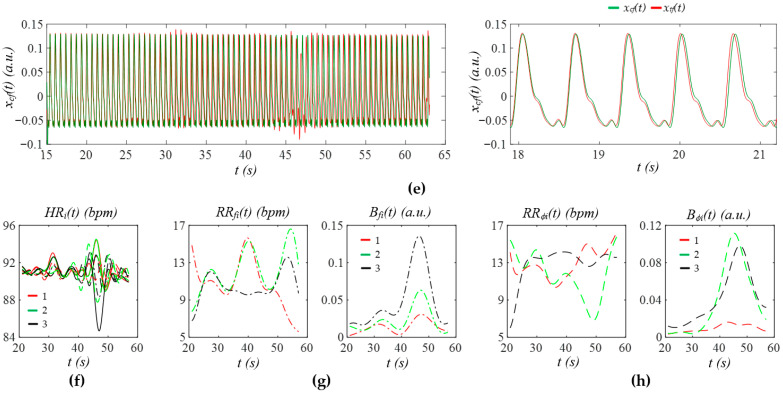
The PPG signal (segment: 150~230 s) of the first critically ill subject (BIDMC dataset): (**a**) original measured pulse signal, *x*_0_(*t*), and *x_b_*(*t*); (**b**) frequency spectrum of *x*_0_(*t*), *x_HVD_*(*t*), *x_tf_*(*t*), *x_cf_*(*t*), and *x_tvsp_*(*t*); (**c**) instant parameters of the harmonics; (**d**) reconstructed pulse signal *x_tf_*(*t*) with *f_i_*(*t*); (**e**) reconstructed pulse signal *x_cf_*(*t*) with *f_C_*; (**f**) HR: *HR_i_*(*t*), *HR_fi_*(*t*), and *HR_ϕi_*(*t*); (**g**) *f_i_*(*t*)-based respiration parameters; and (**h**) *ϕ*_0*i*_(*t*)-based respiration parameters.

Figure 14a shows the PPG signal (segment: 150~230 s) from the first critically ill subject in the dataset BIDMC, revealing a large MA around 47 s. As shown in Figure 14b, the narrow and small sidebands of *x_HVD_*(*t*) and *x_tf_*(*t*) indicate small MA and small HRV. The rapidly changing *x_b_*(*t*) around 47 s is captured by the instant frequency and amplitude noticeably, but is not obvious in the instant initial phase. The respiration signal in the instant initial phase is not as noticeable as in Figure 12c and Figure 13c. As shown in Figure 14d, broadband noise is relatively small and mainly occurs after the upswing in a pulse cycle, affecting the peak, the dicrotic notch, and the notch near the end of a pulse cycle. Similarly, the MA affects the dicrotic notch and the notch near the end of a pulse cycle. As shown in Figure 14e, the notch in *x_tf_*(*t*) varies a bit between pulse cycles, as compared to that in *x_cf_*(*t*). Figure 14f shows that *HR_ϕi_*(*t*) is overall smaller than *HR_i_*(*t*) in amplitude, possibly indicating that respiration has less effect on HR, as compared to the PPG signals in Figure 12 and Figure 13.

**Figure 15:**
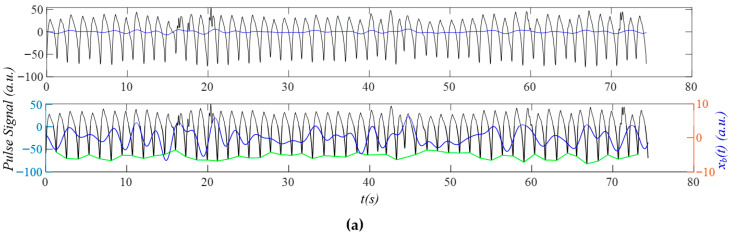

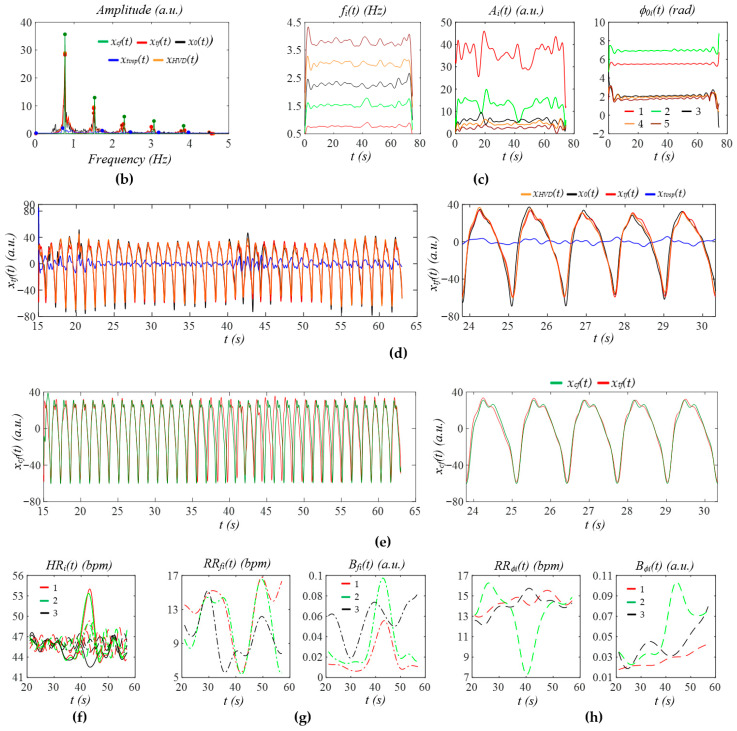
The PPG signal (segment: 150~230 s) of the first subject during acitivties (PPG_DaLiA Dataset): (**a**) original measured pulse signal, *x*_0_(*t*), and *x_b_*(*t*); (**b**) frequency spectrum of *x*_0_(*t*), *x_HVD_*(*t*), *x_tf_*(*t*), *x_cf_*(*t*), and *x_tvsp_*(*t*); (**c**) instant parameters of the harmonics; (**d**) reconstructed pulse signal *x_tf_*(*t*) with *f_i_*(*t*); (**e**) reconstructed pulse signal *x_cf_*(*t*) with *f_C_*; (**f**) HR: *HR_i_*(*t*), *HR_fi_*(*t*), and *HR_ϕi_*(*t*); (**g**) *f_i_*(*t*)-based respiration parameters; and (**h**) *ϕ*_0*i*_(*t*)-based respiration parameters.

Figure 15a shows the PPG signal (segment: 150~230 s) of the first subject from the dataset: PPG DaLiA, revealing rapidly changing *x_b_*(*t*) happening around 20 s and 45 s. Figure 15b shows that the narrow and small sidebands of *x_HVD_*(*t*) and *x_tf_*(*t*) indicate low-level MA and small HRV. As shown in Figure 15c, the rapidly changing *x_b_*(*t*) around 45 s greatly affects the instant amplitude, moderately affects the instant frequency, but does not show any effect on the instant initial phase. However, the rapidly changing *x_b_*(*t*) around 20 s only affects the instant amplitude. The respiration signal in the instant initial phase is also relatively more noticeable than that in Figure 8. As shown in Figure 15d, broadband noise happens primarily near the top and the bottom of a pulse cycle. The small difference between *x_tf_*(*t*) and *x_HVD_*(*t*) indicates a small *x_tvsp_*(*t*). Figure 15e shows that HRV affects the upswing and the notch near the top of a pulse cycle. Figure 15f shows that *HR_ϕi_*(*t*) is overall smaller than *HR_i_*(*t*) in amplitude.

**Figure 16:**
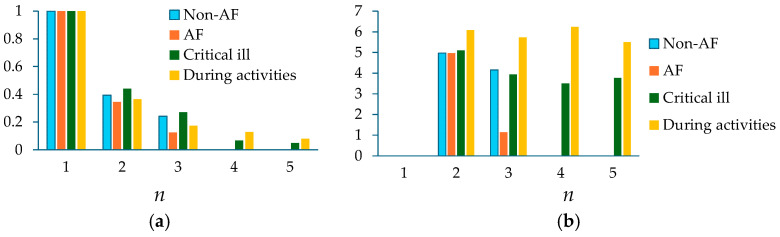
Comparison of *A_cf_i_*/*A_cf__*_1_ and $\bar{\phi }$_0*i*_ − $\bar{\phi }$_01_ of the harmonics of the PPG signals between four physiological conditions: (**a**) *A_cf_i_*/*A_cf__*_1_; (**b**) $\bar{\phi }$_0*i*_ − $\bar{\phi }$_01_.

Table 3 compares the extracted normalized amplitudes and initial phase differences in the harmonics relative to the first harmonic between the four physiological conditions. While $\bar{A}$*_i_*/$\bar{A}$_1_ and *A_cf_i_*/*A_cf__*_1_ are almost identical, *A_x_*_0*i*_/*A_x_*_01_ and *A_tf_i_*/*A_tf__*_1_ are remarkably different, and also are far off from the first two. As shown in Figure 16, the normalized amplitude of the second harmonic is similar between the four PPG signals, but the normalized amplitude of the third harmonic from the AF patient is well below the other three subjects. As compared to the other PPG signals, the PPG signal of the AF patient also shows a much lower initial phase difference in the third harmonic.

Table 4 compares the calculated results on HR and respiration parameters. As compared to the non-AF and during-activities subjects, the HR: mean(*HR_i_*(*t*)) of the AF and critically ill subjects is much higher. As compared to the other subjects, the HRV of the AF subject, RMSE(*HR_i_*(*t*)), is extremely high, providing the reason why *x_b_*(*t*) in his PPG signal varies dramatically with time. In contrast, the HRV of the critically ill subject is even lower than the non-AF and during-activities subjects, explaining why *x_b_*(*t*) in this patient’s PPG signal varies quite moderately with time.

The *f_i_*(*t*)-based RR, mean(*RR_fi_*(*t*)), is lower than the *ϕ_i_*(*t*)-based RR, mean(*RR_ϕi_*(*t*)), for all the subjects, except the non-AF subject. The parameters associated with the *f_i_*(*t*)-based estimation are thus listed but are not used for comparison. Note that the mean(*RR_ϕi_*(*t*)) of the AF and critically ill subjects from one or two harmonics is below 12 Hz. The respiration modulation, mean(*B_ϕi_*(*t*)), is below 0.1 for all the subjects, except the AF subject, whose value is well above 0.1 and indicates a large HRV due to respiration.

The HRV due to respiration, RMSE(*HR_ϕi_*(*t*)), is comparable between the non-AF and during-activities subjects, and is relatively low for the critically ill subject. In contrast, the AF subject reveals an extremely high RMSE(*HR_ϕi_*(*t*)). While RMSE(*HR_PFi_*(*t*)) remains positive for the non-AF and during-activities subjects, it becomes negative in one or two harmonics for the AF and critically ill subjects. As shown in the table, the total HRV (from instant frequency) is not well above the HRV due to respiration (from the instant initial phase). In Section 3.1, it is found that while the instant frequency is slightly affected by MA, the instant initial phase is almost unaffected by MA. As shown in Figure 12, Figure 13, Figure 14 and Figure 15, MA in the AF and critically ill subjects are larger relative to their respective PPG signal, as compared to MA in the non-AF and during-activities subjects. Such a relatively large MA reduces the total HRV to some extent, while the HRV due to respiration remains unaffected by the MA. As such, the reduction in the total HRV caused by a relatively large MA and the large HRV due to respiration leads to negative values of the HRV due to PF. In contrast, as shown in Table 2, since the total HRV is well above the HRV due to respiration, even if MA is large relative to its respective pulse signal, the HRV due to PF will remain positive.

### 3.4. Comparison of Measured Pulse Signals Under Different Physiological Conditions

**Figure 17:**
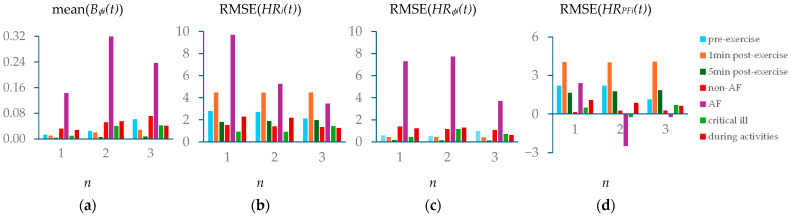
Comparison of extracted parameters from the measured pulse signals: (**a**) respiration modulation; (**b**) total HRV; (**c**) HRV due to respiration; and (**d**) HRV due to PF.

Figure 17 compares respiration modulation: mean(*B_ϕi_(t*)), total HRV: RMSE(*HR_i_*(*t*)), HRV due to respiration: RMSE(Δ*HR_ϕi_*(*t*)), and HRV due to PF: RMSE(*HR_PFi_*(*t*)) between the seven pulse signals. Only the AF subject reveals mean(*B_ϕi_*(*t*)) > 0.1. Furthermore, mean(*B_ϕi_*(*t*) reveals a steadily increasing trend from the first to the third harmonic for the subject under three conditions and the non-AF subject. However, this steadily increasing trend is not observed from the AF, critically ill, and during-activities subjects.

The AF subject also reveals a much higher total HRV, as compared to the rest subjects. Note that the high total HRV for the subject after 1 min post-exercise is due to the post-exercise recovery of the heart. The total HRV remains at similar levels between the three harmonics for the subject under three conditions and the non-AF subject, but not for the other three subjects. The AF subject especially reveals a decreasing trend from the first to the third harmonic on total HRV.

The AF subject reveals an extremely high HRV due to respiration, as compared to the rest subjects. Moreover, HRV due to respiration remains at similar levels between the three harmonics for the subject under three conditions and the non-AF subject, but varies noticeably for the other three subjects. Similarly, HRV due to PF remains at similar levels between the three harmonics for the subject under three conditions and the non-AF subject, but varies noticeably for the other three subjects. HRV due to PF falls into negative territories in one or two harmonics for the AF and critically ill subjects, as explained earlier. The high HRV due to PF for the subject after 1 min post-exercise reveals the role of PF in regulating HR during the post-exercise recovery.

Among the three measured pulse signals using a tactile sensor, the signal pre-exercise encounters relatively large broadband noise. Yet, as compared to the measured pulse signals using a tactile sensor, the PPG signals encounter much larger broadband noise, as evidenced by the large differences between *x_HVD_*(*t*) and *x*_0_(*t*) in Figure 12, Figure 13 and Figure 15, except for the PPG signal in Figure 14, which reveals small broadband noise. From Table 2 and Table 4, it can be seen that the pulse signals with large broadband noise also exhibit large total HRV. Note that the pulse signal 1 min post-exercise encounters a large HRV but low broadband noise, because its large HRV arises from the post-exercise recovery of the heart, not the inherent HRV at rest. Thus, except for the pulse signal 1 min post-exercise, broadband noise in all the rest pulse signals heavily depend on HRV and by extension MA, since MA follows the multiple sidebands of HRV.

### 3.5. Comparison of Measured Pulse Signals Between at Rest and During Activities 

The PPG signal in Figure 15 is measured at the wrist during activities. Its extracted APW differs greatly from the one in Figure 8, which is typical of the APW at the wrist for young healthy subjects at rest pre-exercise: steep upswing and the dicrotic notch around the middle of the APW. The MA during activities falls into the frequency range of the pulse signal itself [10,20]. As shown in Figure 6 and Figure 7, MA during activities can not only greatly affect the instant amplitude and instant initial phase of the harmonic whose frequency coincides with or is close to the frequency of this MA, but also add distortion to other harmonics whose frequency is close to the sidebands of this MA. Additionally, there might be two other reasons: (1) the tissue–sensor contact is loose and thus the values of *m*_0_, *k*_0_, and *c*_0_ of the TCS stack are totally different during activities; and (2) the true pulse signal at the wrist during activities might differ from the one at rest. Nevertheless, it may be concluded that a measured pulse signal during activities may be suitable for the extraction of HR and respiration parameters, but unsuitable for the extraction of APW. Finally, it must be noted that activities can easily cause intermittent tissue–sensor contact, severely distorting pulse cycles and causing large errors in extracted HR and respiration parameters [10].

## 4. Discussion

### 4.1. Qualitative Validation of the Generalized SDOF-TF Method

As to the measured pulse signals of one subject using a tactile sensor under three conditions, as compared to pre-exercise, the observed differences include the following: (1) the dicrotic notch is greatly lowered by exercise; (2) the HRV is reduced 5 min post-exercise; (3) the HR decreases rapidly 1 min post-exercise. These observed differences are consistent with the related findings using medical instruments in the literature [1,3,47,48]. The observed change in extracted APW is that the normalized amplitudes of the second and third harmonics are increased and decreased by exercise, respectively, and are consistent with the findings at the neck from the measured pulse signals on a healthy 32-yr-old male subject using the same sensor [21].

AF refers to irregular HR, and the characteristics of AF are large HRV [10,49]. Comparison of the PPG signal of the AF subject with that of the rest subjects reveals the large HRV of the AF subject. Moreover, this generalized SDOF-TF method also reveals that this large HRV (1) primarily comes from respiration and (2) decreases with the harmonic order. Although respiration modulation and the normalized amplitude and relative initial phase difference in the third harmonic of the AF patient are found to greatly differ from their counterparts of the other three subjects, no related findings are reported in the literature. Thus, the analysis of the PPG signals from more subjects is needed to establish statistical significance for these three arterial indices for AF detection. Nevertheless, some of the observed differences between physiological conditions are consistent with the related findings, qualitatively validating the effectiveness of the SDOF-TF method in MA removal from a measured pulse signal at rest and extraction of APW, HR, and respiration parameters, regardless of the sensor type and physiological conditions (or different MA).

### 4.2. The Effect of MA and HRV on Extracted APW, HR, and Respiration Parameters

**Figure 18:**
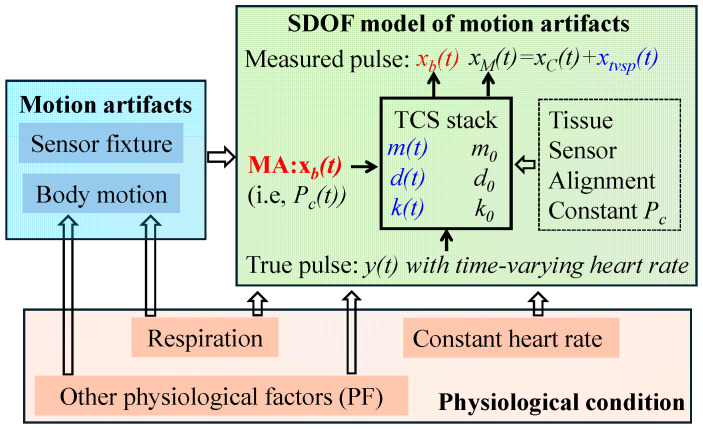
An SDOF model of MA with all the factors in arterial pulse measurement using a PPG sensor (note: for a tactile sensor, replace MA: *x_b_*(*t*) with MA: *z_b_*(*t*) and add *k_s_* and *d_s_* to the TCS stack).

Figure 18 summarizes how physiological condition and MA are entangled in a measured pulse signal. On the one hand, physiological condition dictates respiration and PF, and it leads to time-varying heart rate (i.e., HRV). On the other hand, physiological condition dictates body motion. MA, as a combination of body motion and sensor fixture, cause baseline drift *x_b_*(*t*). While tissue, sensor, sensor alignment, and constant *P_c_* presets the nominal values of the SDOF system parameters *m*_0_, *d*_0_, *and k*_0_, for the TCS stack, baseline drift *x_b_*(*t*) (i.e., time-varying *P_c_*(*t*)) causes TVSP: *m*(*t*), *d*(*t*), and *k*(*t*), in the TCS stack, and further TVSP-generated distortion *x_tvsp_*(*t*) in a measured pulse signal. Since *x_tvsp_*(*t*) rides on each harmonic of the true pulse signal, *x_tvsp_*(*t*) captures physiological conditions twice: time-varying heart rate and TVSP. As such, *x_tvsp_*(*t*) heavily depends on physiological condition or HRV. As shown in Figure 13, the large HRV of the AF subject causes a large *x_tvsp_*(*t*), as compared to all the rest subjects.

Besides MA and HRV (respiration and PF), sensor noise and nonlinearity of the TCS stack [7,8,34] may also affect a measured pulse signal. As shown in Section 3.4, MA, HRV, and sensor noise are not independent of each other. HRV determines the multiple sidebands of MA and consequently determines broadband noise. Although respiration affects both MA (in terms of baseline drift) and HR, for simplicity, the effect of respiration on a measured pulse signal is solely attributed to HR in this discussion. Since there is no established theory on sensor noise, and nonlinearity is commonly considered to be small and negligible as long as the tissue–sensor contact remains stable [21], only the effect of MA and HRV on a measured pulse signal is discussed here. Given that PF affects HRV, the effect of respiration on the instant frequency obtained in Section 3.1 is directly extended to HRV.

MA in a measured pulse signal heavily depends on physiological conditions (i.e., HRV) of a subject, simply because HRV determines the multiple sidebands of each harmonic, and yet *x_tvsp_*(*t*) stemming from MA (or baseline drift *x_b_*(*t*)) rides on each harmonic of the true pulse signal and thus rides on these multiple bands. The SDOF-TF method allows for distinguishing the effect of HRV from the effect of MA on a measured pulse signal. On the one hand, MA (via *x_tvsp_*(*t*)) causes great swings in the instant amplitude of each harmonic in a measured pulse signal, but affects the instant frequency to a very limited extent, and has no effect on the straightness of the instant initial phase. On the other hand, the effect of HRV on instant amplitude is very small, but is clearly manifested in instant frequency, and respiration is clearly manifested in the instant initial phase. Given that the slope of the instant initial phase in the Hilbert transform is not well defined, the instant initial phase can be used only for the extraction of respiration parameters due to its time-harmonic nature, but not PF. The difference in HRV from instant frequency and HRV from the instant initial phase can then be used as HRV due to PF.

Due to the slight effect of MA on instant frequency, respiration parameters extracted from instant frequency carry more errors, as compared to their counterparts extracted from the instant initial phase. In contrast, instant initial phase extracts respiration parameters with better accuracy, due to its much larger immunity to MA. It should be noted that the instant initial phase is subject to sensor noise. As shown in Figure 8c, the abnormal instant amplitude of the third harmonics is believed to arise from sensor noise, which might further affect the instant initial phase.

The extracted APW with HRV (or time-varying frequency) varies between pulse cycles. A large HRV can cause great variation in APW between pulse cycles. Conversely, a small HRV will cause a small variation in APW between pulse cycles. Given that current arterial indices based on APW rely on the fine features of the APW itself and its time derivatives. Even a small variation in APW may greatly influence the values of the derived arterial indices. As shown in Section 3, the extracted APW with constant HR eliminates the effect of HRV on APW. The amplitudes and initial phases of the harmonics in this extracted APW greatly differ from their counterparts with HRV and are believed to serve better as arterial indices for comparison between physiological conditions. As shown in Section 3, the dicrotic notch being lowered by exercise might be partially due to the changes in the normalized amplitudes of the second and third harmonics.

MA during activities falls into the frequency range of the true pulse signal, and its frequency is unknown and may vary between pulse cycles. As shown in Section 3, the effect of MA during activities on each harmonic of a measured pulse signal is unpredictable if the frequency of this MA is unknown. Moreover, activities can easily break tissue–sensor contact and severely distort pulse cycles [10]. As such, accurate extraction of HR and RR from a PPG signal during activities might not be practical.

### 4.3. Comparison with the Related Studies

Here, this SDOF-TF method is compared to the related studies based on time–frequency analysis: VFCDM, DWT, and EMD. It should be noted that all these methods have focused on PPG signals. EMD encounters mode mixing due to the close proximity in frequency between the harmonics [16,19]. DWT needs pre-defined wavelet functions for MA [19]. As shown here, MA varies between physiological conditions. As such, DWT needs to tailor its wavelet functions to each physiological condition.

The VFCDM method is generalized in the sense that it does not need any pre-defined wavelet functions. This method uses a bandpass filter to separate the harmonics in a PPG signal, and mode mixing does not occur. Initially, this method used instant frequency to extract RR only [16]. Recently, this method was used to detect AF using PPG signals measured at the wrist during activities [10]. It first identifies a clean segment (i.e., low-level MA) of pulse cycles in a PPG signal via their frequency spectrum. The recorded acceleration signal is further used to remove any falsely identified clean segments. MA in these pulse cycles are neglected, instead of being removed. HR is extracted in the time domain (i.e., peak-to-peak interval) and HRV is calculated. An overlap in HRV between AF and non-AF subjects from two datasets is observed [10].

This study extracts HR from the instant frequency. The calculated HRV based on it shows a clear difference between the AF subject and the rest subjects from the first and second harmonics. Yet, the extracted respiration modulation from the instant initial phase and the extracted HRV due to respiration show an even clearer difference from all three harmonics. Another identified difference is from the extracted APW: the normalized amplitude and relative initial phase difference in the third harmonic are lower in the AF subject. In the future, the PPG signals of a large number of subjects need to be analyzed to establish statistical significance for these new indices.

Finally, none of these three methods have explored the instant initial phase of a harmonic for the extraction of RR and respiration modulation, as well as the separation of HRV between respiration and PF. It must be admitted that the extracted HRV from instant frequency (or total HRV) may carry errors from MA, nonlinearity, and sensor noise. However, it is believed that the extracted RR, respiration modulation, and HRV due to respiration from the instant initial phase carry primarily errors from sensor noise. As compared to the related studies in the literature, the key findings from this study can be summarized as follows:

1. The effects of MA at rest and respiration on a measured pulse signal are different: (1) the MA affects a measured pulse signal via its effect on the TCS stack (or *x_tvsp_*(*t*)), and (2) respiration affects a measured pulse signal via its effect on HR. Their effects on the instant parameters of each harmonic in a measured pulse signal are different.
2. The effect of MA at rest on a measured pulse signal can be removed via the regression line of instant amplitude and the mean of instant initial phase.
3. As compared to instant frequency, instant initial phase is almost immune to MA and nonlinearity, provides the extraction of respiration parameters and HRV due to respiration with better accuracy, and better distinguishes the HRV due to respiration from the HRV due to PF.
4. A large HRV (or a large time-varying frequency) affects the APW (amplitude and initial phase of each harmonic) to a great extent. The reconstructed pulse signal with constant HR serves better as the extracted APW to derive the amplitude and initial phase of each harmonic for comparison between physiological conditions.
5. A large HRV translates to large multiple sidebands of MA (or *x_tvsp_*(*t*)) and, by extension, large broadband noise, given the same sensor.
6. Due to the unpredictability of MA during activities, in terms of its frequency, MA in a measured pulse signal during activities is difficult to detect and remove.

### 4.4. Study Limitation

There are five main study limitations. Firstly, in the analysis of all the measured pulse signals, the nonlinearity of the TCS stack is neglected. Given the geometrical and anatomical complexity of the tissue above an artery and its nonlinear and viscoelastic properties, some nonlinearity in the TCS stack is expected. However, contact pressure for establishing the tissue–sensor contact greatly alleviates such nonlinearity. This is evidenced by a smooth arterial pulse signal with no abrupt changes in its waveform. As far as the tissue–sensor contact is stable (not intermittent), this nonlinearity is believed to be small [21]. As shown here, the analyzed results in Section 3 without considering nonlinearity yield the differences between physiological conditions that are consistent with the related findings in the literature. As such, the assumption of a linear TCS stack is practical. In contrast, MA during activities may cause intermittent tissue–sensor contact, which introduces great nonlinearity in a measured pulse signal, as evidenced by abrupt changes in the measured pulse waveform [10]. Note that the pulse segments from all the PPG signals are chosen with small MA, since a segment with large MA is believed to encounter unstable tissue–sensor contact and then suffer non-negligible nonlinearity in the TCS stack.

Secondly, the complexity of optical transduction in a PPG sensor is greatly simplified. Similarly to nonlinearity, such great simplification does not obscure the expected difference in HRV between the AF subject and the other subjects. Thirdly, sensor noise is not considered. As shown in Figure 8c, the abrupt change in *A_i_*(*t*) of the third and fourth harmonic pre-exercise indicates the existence of sensor noise.

Fourthly, the analyzed results lack statistical significance between physiological conditions. Although some observed differences between physiological conditions are validated using the related findings in the literature, there are no studies reported on the differences in (1) respiration modulation of the three harmonics, (2) normalized amplitude, and (3) relative initial phase difference in the third harmonic between AF and non-AF. As such, all the PPG signals of AF and non-AF subjects in the dataset need to be analyzed to establish the statistical significance of these arterial indices in the future. Lastly, a quantitative comparison of this SDOF-TF method with the existing methods, such as DWT and EMD, is not conducted. Such a quantitative comparison will become possible when the SDOF-TF method is applied to unified datasets of measured pulse signals for statistical significance.

Again, this study focuses on examining and validating the effectiveness and generalizability of the SDOF-TF method for analyzing any measured pulse signals at rest, and providing a thorough examination of the effects of MA and respiration (or HRV) on a measured pulse signal and its extracted APW, HR, and respiration parameters. Thus, measured pulse signals using different sensors under different physiological conditions are analyzed, instead of analyzing measured pulse signals of a large number of subjects with the same physiological condition using the same sensor for statistical significance.

In recent years, deep learning has been intensively applied to PPG signals for MA removal and extraction of HR [50,51,52,53]. Yet, it works somehow like a black box and lacks interpretability and transparency, hindering clinical trust and adoption [50,51,52,53]. In contrast, the SDOF-TF method provides great interpretability on how MA is related to a TCS stack and physiological condition and how MA (i.e., *x_tvsp_*(*t*)) alters the measured pulse signal, without the need to use any references. As detailed in Section 3, the analysis of the seven measured pulse signals under different physiological conditions sheds unambiguous insights into the effect of different levels of MA on a measured pulse signal, providing all the necessary details for its transparency and interpretability.

## 5. Conclusions

In this paper, a generalized SDOF-TF method is presented that allows for reconstruction of a measured pulse signal free of MA and extraction of APW, HR, and respiration parameters, regardless of the sensor type and physiological conditions, as long as the pulse signal is measured at rest. This method is built upon an SDOF model of MA, which distinguishes the effects of MA and respiration on the instant parameters: instant amplitude, instant frequency, and instant initial phase of each harmonic in a measured pulse signal. This method has been applied to measured pulse signals at rest using a tactile sensor and PPG signals under different physiological conditions, and is qualitatively validated with the related findings in the literature.

With this method, the effects of MA and respiration (or HRV) on these measured pulse signals, reconstruction of a pulse signal free of MA, and extraction of APW, HR, and respiration parameters are thoroughly examined. It is found that the instant initial phase of a harmonic provides the extraction of respiration parameters with better accuracy, as compared to instant frequency. HRV between respiration and PF can be separated. Because HRV (or time-varying frequency) causes APW variation between pulse cycles, the reconstructed pulse signal with constant HR is treated as the extracted APW, which serves better to extract the amplitudes and initial phases of the harmonics in a measured pulse signal for comparison between physiological conditions. In the future, this method will be applied to measured pulse signals of a large number of subjects for establishing the statistical significance of their extracted parameters.

## Figures and Tables

**Table 1:** Normalized amplitudes and initial phase difference in the harmonics relative to the 1st harmonic of the measured pulse signals at the wrist using a tactile sensor.

	*n*	${\bar{A}}_{i}/\bar{A}$ _1_	*A_x_*_0*i*_/*A_x_*_01_	*A_tf_i_*/*A_tf__*_1_	*A_cf_i_*/*A_cf__*_1_	$\bar{\phi }$_0*i*_ − $\bar{\phi }$_01_
pre-exercise	1	1	1	1	1	0
	2	0.457	0.324	0.332	0.457	5.781
	3	0.407	0.209	0.219	0.407	5.420
	4	0.231	0.128	0.114	0.231	4.460
	5	0.146	0.085	0.071	0.146	3.864
	6	0.115	0.067	0.070	0.115	3.716
	7	0.066	0.033	0.036	0.065	2.854
1 min post-exercise	1	1	1	1	1	0
	2	0.698	0.571	0.515	0.698	4.954
	3	0.261	0.147	0.151	0.261	4.335
	4	0.156	0.088	0.088	0.155	3.380
	5	0.074	0.040	0.032	0.073	2.501
	6	0.032	0.015	0.014	0.032	2.005
	7	0.016	0.008	0.006	0.016	3.004
5 min post-exercise	1	1	1	1	1	0
	2	0.612	0.524	0.541	0.611	5.082
	3	0.237	0.151	0.154	0.236	4.060
	4	0.127	0.072	0.070	0.126	3.408
	5	0.043	0.023	0.028	0.042	3.011
	6	0.010	0.008	0.007	0.010	3.003
	7	0.010	0.007	0.001	0.010	3.104

**Table 2:** Calculated HR, respiration parameters, and associated HRV of the measured pulse signals at the wrist using a tactile sensor.

	Pre-Exercise			1 min Post-Exercise			5 min Post-Exercise		
Harmonics (*n*)	1	2	3	1	2	3	1	2	3
mean(*HR_i_*(*t*))	61.88	61.88	61.48	127.18	127.20	127.19	102.12	102.12	102.11
RSME(*HR_i_*(*t*))	2.78	2.72	2.12	4.48	4.47	4.48	1.81	1.89	1.97
mean(*RR_fi_*(*t*))	10.14	10.01	7.02	7.27	7.29	7.21	6.29	6.32	6.30
mean(*B_fi_*(*t*))	0.029	0.055	0.092	0.032	0.063	0.092	0.027	0.061	0.099
mean(*HR_fi_*(*t*))	60.65	60.74	60.85	127.76	127.76	127.75	102.10	102.14	102.12
mean(*RR_ϕi_*(*t*))	14.92	14.74	13.44	13.43	13.39	13.35	12.58	12.36	12.40
mean(*B_ϕi_*(*t*))	0.013	0.024	0.062	0.010	0.020	0.028	0.004	0.006	0.007
mean(*HR_ϕi_*(*t*))	60.72	60.71	60.73	127.73	127.73	127.73	102.15	102.14	102.14
RMSE(*HR_ϕi_*(*t*))	0.58	0.53	0.97	0.43	0.45	0.42	0.16	0.13	0.12
RMSE(*HR_PFi_*(*t*))	2.20	2.19	1.14	4.04	4.02	4.06	1.65	1.76	1.85

**Table 3:** Normalized amplitudes and initial phase differences in the harmonics relative to the 1st harmonic of the four PPG signals in Figure 12, Figure 13, Figure 14 and Figure 15.

	Non-AF			AF			Critically Ill					During Activities				
*n*	1	2	3	1	2	3	1	2	3	4	5	1	2	3	4	5
$\bar{A}$*_i_*/$\bar{A}$*_i_*	1	0.393	0.241	1	0.345	0.123	1	0.440	0.270	0.066	0.049	1	0.363	0.172	0.128	0.080
*A_x_*_0*i*_/*A_x_*_01_	1	0.470	0.203	1	0.287	0.075	1	0.290	0.186	0.045	0.029	1	0.255	0.104	0.083	0.035
*A_tf_i_*/*A_tf__*_1_	1	0.260	0.186	1	0.280	0.048	1	0.379	0.187	0.036	0.028	1	0.321	0.122	0.089	0.044
*A_cf_i_*/*A_cf__*_1_	1	0.393	0.241	1	0.344	0.124	1	0.439	0.269	0.066	0.049	1	0.363	0.172	0.127	0.080
$\bar{\phi }$_0*i*_ − $\bar{\phi }$_01_	0	4.972	4.156	0	4.973	1.140	0	5.099	3.940	3.497	3.766	0	6.080	5.725	6.239	5.503

**Table 4:** Calculated HR, respiration parameters, and associated HR variations from the four PPG signals in Figure 12, Figure 13, Figure 14 and Figure 15.

	Non-AF			AF			Critically Ill			During Activities		
*n*	1	2	3	1	2	3	1	2	3	1	2	3
mean(*HR_i_*(*t*))	59.09	59.11	59.12	97.97	94.08	90.98	91.11	91.10	90.69	45.86	45.87	45.04
RMSE(*HR_i_*(*t*))	1.52	1.40	1.35	9.69	5.25	3.47	0.94	0.91	1.41	2.28	2.17	1.26
mean(*RR_fi_*(*t*))	12.56	12.59	12.58	9.38	10.55	10.36	10.76	12.19	10.54	12.71	11.31	9.83
mean(*B_fi_*(*t*))	0.027	0.039	0.057	0.143	0.186	0.109	0.014	0.023	0.050	0.019	0.034	0.055
mean(*HR_fi_*(*t*))	58.95	58.94	58.94	94.63	95.57	95.43	90.97	90.95	90.94	46.13	46.11	45.90
mean(*RR_ϕi_*(*t*))	12.58	12.54	12.47	10.63	12.46	11.05	12.82	11.58	12.98	14.35	13.16	14.06
mean(*B_ϕi_*(*t*))	0.032	0.052	0.071	0.143	0.319	0.237	0.010	0.040	0.042	0.028	0.055	0.041
mean(*HR_ϕi_*(*t*))	58.89	58.89	58.91	95.66	95.14	95.30	90.97	90.96	90.97	45.94	45.94	46.04
RSME(*HR_ϕi_*(*t*))	1.38	1.16	1.07	7.30	7.74	3.71	0.44	1.15	0.71	1.21	1.29	0.61
RMSE(*HR_PFi_*(*t*))	0.13	0.25	0.27	2.39	−2.49	−0.24	0.50	−0.24	0.70	1.07	0.88	0.64

## Data Availability

The data on the measured pulse signals using a tactile sensor that support the findings of this study are available upon reasonable request from the author. The data are not publicly available due to privacy or ethical restrictions.

## Abbreviations

The following abbreviations are used in this manuscript:

APW	Arterial pulse waveform
MA	Motion artifacts
TVSP	Time-varying system parameters
SDOF	Single degree of freedom
SDOF-TF	Single degree of freedom time–frequency
HVD	Hilbert vibration decomposition
HR	Heart rate
HRV	Heart rate variability (in terms of RMSE)
RR	Respiration rate
PF	Physiological factors excluding respiration on heart rate regulation
